# Head-to-tail polymerization by VEL proteins underpins cold-induced Polycomb silencing in flowering control

**DOI:** 10.1016/j.celrep.2022.111607

**Published:** 2022-11-08

**Authors:** Marc Fiedler, Elsa Franco-Echevarría, Anna Schulten, Mathias Nielsen, Trevor J. Rutherford, Anna Yeates, Bilal Ahsan, Caroline Dean, Mariann Bienz

**Affiliations:** 1 MRC Laboratory of Molecular Biology, Francis Crick Avenue, Cambridge CB2 0QH, UK; 2 John Innes Centre, Norwich Research Park, Norwich NR4 7UH, UK

**Keywords:** head-to-tail polymerization, biomolecular condensation, atypical four-helix bundle, DNAJ co-chaperones, Polycomb silencing, inheritance of repressed state, cold-induced flowering control

## Abstract

Transcriptional silencing through the Polycomb silencing machinery utilizes a “read-write” mechanism involving histone tail modifications. However, nucleation of silencing and long-term stable transmission of the silenced state also requires P-olycomb Repressive Complex 2 (PRC2) accessory proteins, whose molecular role is poorly understood. The *Arabidopsis* VEL proteins are accessory proteins that interact with PRC2 to nucleate and propagate silencing at the *FLOWERING LOCUS C* (*FLC*) locus, enabling early flowering in spring. Here, we report that VEL proteins contain a domain related to an atypical four-helix bundle that engages in spontaneous concentration-dependent head-to-tail polymerization to assemble dynamic biomolecular condensates. Mutations blocking polymerization of this VEL domain prevent Polycomb silencing at *FLC*. Plant VEL proteins thus facilitate assembly of dynamic multivalent Polycomb complexes required for inheritance of the silenced state.

## Highlights

• VEL proteins assemble biomolecular condensates by head-to-tail polymerization
• Their polymerizing fold is an atypical four-helix bundle also found in co-chaperones
• Polymerization of VEL proteins assists transcriptional silencing by Polycomb
• VEL polymerization is required for the cold-induced transition to flowering

## Graphical abstract

**Figure:**
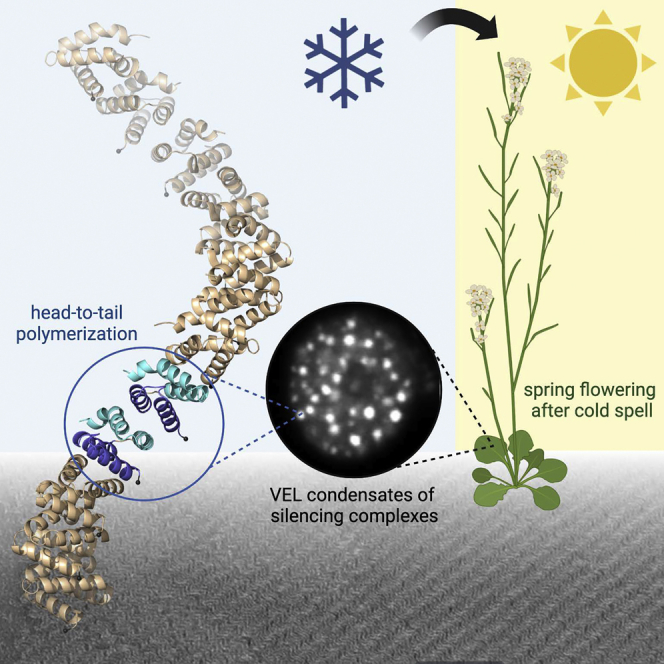

## Teaser

Fiedler et al. report that VEL proteins assemble biomolecular condensates by head-to-tail polymerization to promote early flowering in spring. Their polymerizing domain resembles an atypical four-helical bundle akin to those found in universal co-chaperones. VEL polymerization assists the Polycomb machinery in the sustained silencing of a flowering control locus.

## Introduction

Transcriptional silencing by the Polycomb system is central to the development of all multicellular organisms, but the mechanisms that nucleate this process and ensure its inheritance through multiple cell divisions remain poorly understood. Its hallmark is the tri-methylation of lysine 27 on the tail of histone H3 (H3K27me3) imposed on Polycomb target genes by the highly conserved Polycomb Repressive Complex 2 (PRC2) (Margueron and Reinberg, 2011; Bieluszewski et al., 2021; Chittock et al., 2017). This chromatin mark needs to be initiated at specific sites in target genes, known as Polycomb Response Elements (PREs) in *Drosophila*, and then propagated through numerous cell divisions to maintain cell identities (Simon, 1995). Nucleation of Polycomb silencing at specific sites requires accessory proteins, and propagation of H3K27me3 involves a “read-write” mechanism: PRC2 not only delivers H3K27me3 by virtue of its methyltransferase activity but also binds to H3K27me3 through a cognate domain of one of its subunits, which enables this complex to deposit the same mark on nearby unmodified nucleosomes (Margueron et al., 2009; Hansen et al., 2008; Margueron and Reinberg, 2011). This is thought to restore H3K27me3 to both daughter DNA strands after each round of replication. However, several recent studies have suggested that this read-write mechanism alone is insufficient to maintain robust inheritance when replication rates are high (Coleman and Struhl, 2017; Laprell et al., 2017). Accessory proteins are required to ensure robust inheritance of silencing.

**Figure 1:**
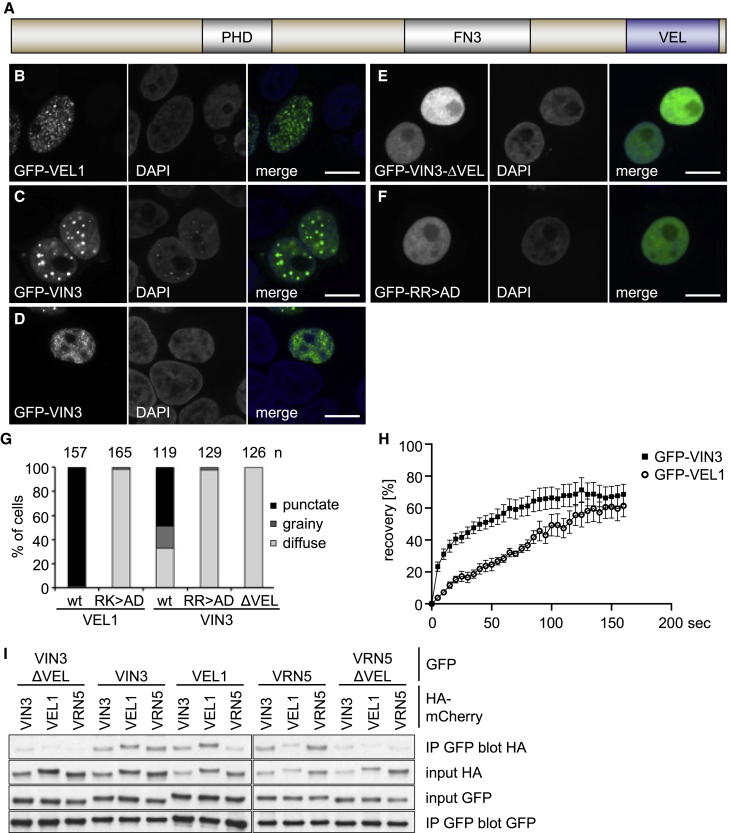
VEL-dependent nuclear condensation of VEL proteins (A) Domain architecture of VIN3. (B–F) Representative confocal images of HeLa cells transfected with WT or mutant GFP-VIN3 or GFP-VEL1 (green in merges), after fixation and staining with DAPI (blue in merges) to mark nuclei, as indicated in panels; scale bars: 10 μm. (G) Quantitation of protein distributions in (B)–(E), whereby grainy fluorescence (D) was typically observed at lower expression levels (see also STAR methods); n, numbers of cells scored. (H) FRAP of GFP-VIN3 (squares) or GFP-VEL1 (circles) of >5 medium-sized nuclear puncta acquired from 3–6 different HeLa cells; error bars, standard error of the mean (SEM). (I) coIP of WT and mutant tagged VEL proteins, as indicated, upon co-overexpression in HEK293T cells. See also Figure S1.

One well-studied PRC2 target gene in *Arabidopsis* is the *FLOWERING LOCUS C* (*FLC*). *FLC* is progressively silenced through winter by a low-probability digital, cell-autonomous on/off switching mechanism, a process known as vernalization (Angel et al., 2011; Berry et al., 2015; Henderson and Dean, 2004; Ruelens et al., 2013; Whittaker and Dean, 2017). Arabidopsis PRC2 interacts with accessory factors including the VEL protein family, with two members, VIN3 and VRN5 (also known as VIL1), shown genetically to be required for vernalization (Sung and Amasino, 2004; Greb et al., 2007). Their hallmarks are an atypical plant homeodomain (PHD) zinc finger and a fibronectin type III (FN3) domain, plus a C-terminal VEL domain of unknown structure and function (Greb et al., 2007; Sung and Amasino, 2004; Wood et al., 2006) (Figure 1A). Cold induces expression of VIN3, while VRN5 and VEL1 are constitutively expressed (Greb et al., 2007; Sung et al., 2006). VEL proteins associate with PRC2 at *FLC* dynamically during initiation of silencing at a three-nucleosome nucleation region; this results in the delivery of localized H3K27me3, which then spreads across the locus after transfer back to warm temperatures (Yang et al., 2017). Genetic disruption of the H3K27me3 spreading at *FLC* revealed that nucleation alone holds metastable silencing for ∼10 cell cycles; however, modeling indicated that the H3K27me3 read-write mechanism is insufficient to maintain metastable silencing over this length of time. We thus proposed that persistence of memory at the *FLC* locus requires additional factors capable of clustering into assemblies that can persist through DNA replication (Lövkvist et al., 2021). Likely candidates for these factors are the VEL proteins as their ability to engage in homo- and mutual hetero-typic interactions may allow them to self-assemble into such clusters (Greb et al., 2007; Sung et al., 2006).

Here, we report that purified VEL domains undergo spontaneous head-to-tail polymerization *in vitro*, which enables them to assemble dynamic biomolecular condensates in cell nuclei. Crystallography and nuclear magnetic resonance (NMR) uncovered an ancient globular fold composed of four α-helices, which engages in spontaneous head-to-tail interactions to form short protofilaments and striking higher-order superstructures. This is only the third such head-to-tail polymerization fold to be described, adding to the sterile alpha motif (SAM) and Disheveled and Axin (DIX) domains, whose folds are structurally distinct. These mediate assembly of biomolecular condensates to facilitate diverse processes such as transduction of Wnt and other signals, transcription, and RNA processing (Bienz, 2020; Qiao and Bowie, 2005; Schwarz-Romond et al., 2007; note that the DIX domain family also contains the closely related PB1 domains; Bienz, 2014; Bienz, 2020). Our results from functional complementation assays in mutant *Arabidopsis* plants indicate that VEL-dependent head-to-tail polymerization is required for cold-induced Polycomb silencing underpinning flowering control.

## Results

### VEL-dependent nuclear condensation of VEL proteins

We had previously found that the VEL proteins form nuclear speckles in plant cells that depended on their VEL domains (Greb et al., 2007). We therefore asked whether they would also cluster in heterologous mammalian cells in the absence of endogenous plant proteins. This is indeed the case: wild-type (WT) GFP-VIN3 and GFP-VEL1 consistently produce discrete puncta in the nuclei of transiently transfected HeLa (Figures 1B and 1C) or COS-7 cells (Figure S1), although a minority of cells showed grainy GFP-VIN3, typically seen at lower expression levels (Figure 1D). In contrast, deletion mutants without their VEL domains (GFP-VIN3_ΔVEL_ or GFP-VEL1_ΔVEL_) or double-point mutants whose self-assembly is blocked (see below), named RR>AD (VIN3) or RK>AD (VEL1), are always diffuse (Figures 1E and 1F), as is WT GFP-VRN5 (Figure S1). A quantification of these patterns is shown in Figure 1G. To validate our results obtained in these mammalian cell-based assays, we also expressed WT or mutant GFP-VIN3 proteins in transiently transfected *Nicotiana benthamiana* (tobacco) leaves, which confirmed that WT GFP-VIN3 is punctate in every single transfected cell, whereas the mutants show a diffuse pattern (n = 10; Figure S1). Note that the expression levels of these mutants are comparable to those of the WT protein (Figure S1; see also below). We conclude that GFP-VIN3 and GFP-VEL1 each exhibits an inherent capability to form punctate nuclear structures, regardless of the cellular expression system. We therefore focused on these two VEL proteins for subsequent characterization of their puncta-forming activity.

Fluorescence recovery after photobleaching (FRAP) assays revealed that the GFP-VIN3 and GFP-VEL1 puncta are highly dynamic: the former recover <70% of the initial fluorescence with a half-time (t_1/2_) of 8–10 s, whereas GFP-VEL1 puncta are somewhat less dynamic (t_1/2_: 70 s) (Figure 1H). This indicates a rapid exchange between the nuclear punctate and diffuse pools of both proteins, similar to that observed for other biomolecular condensates (Banjade and Rosen, 2014; Bienz, 2014), including the nuclear speckles formed in mouse embryonic cells by SAM-dependent polymerization of Polyhomeotic C2 proteins, which exhibit FRAP profiles similar to those of GFP-VEL1 (Isono et al., 2013).

Previous yeast two-hybrid (Y2H) assays had revealed that VIN3 and VEL1 self-interact and that VIN3 also cross-interacts with VRN5 and VEL1 (Greb et al., 2007; Sung et al., 2006). Using co-immunoprecipitation (coIP) assays in transfected human embryonic kidney (HEK293T) cells, we confirmed these patterns of self- and cross-association (Figure 1I; we also confirmed that VRN5 can self-interact). Importantly, in both assays, self- and cross-interactions between VEL paralogs depend on their VEL domains (Figure 1I), as indicated by previous studies (Greb et al., 2007; Sung et al., 2006). Thus, the VEL-dependent self-association of these VEL proteins is likely to confer their dynamic clustering in cell nuclei.

### Concentration-dependent polymerization by VEL domains

**Figure 2:**
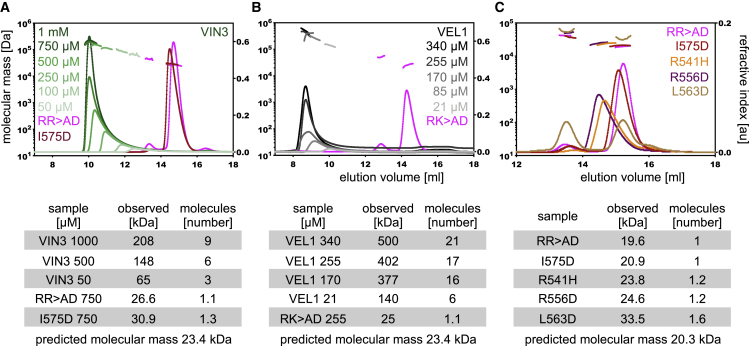
Concentration-dependent polymerization by VEL domains SEC-MALS of purified WT or mutant (A and C) Lip-VIN3_VEL_ (residues 500–603) or (B) Lip-VEL1_VEL_, at increasing concentrations as indicated in panels; curves, elution profiles (void volume of column at 8 mL); line traces, molar masses as derived from MALS; these are specified in the tables below panels, which also indicate numbers of molecules per oligomer at a given concentration. See also Figure S2.

Next, we asked whether these properties of the VEL domains may reflect their ability to undergo head-to-tail polymerization. We thus purified recombinant VIN3_VEL_ (residues 500–603) and VEL1_VEL_ (residues 589–692) bearing a lipoyl (Lip) solubility tag after expression in *Escherichia coli* to conduct size-exclusion chromatography coupled with multiangle light scattering (SEC-MALS). Indeed, both WT domains self-associate to form short polymers in a concentration-dependent fashion, reaching ∼9-mer (VIN3_VEL_) or >20-mer (VEL1_VEL_) at high concentrations (Figures 2A and 2B), indicating their ability to polymerize. The same is true for Lip-tagged domains purified from VEL orthologs found in distantly related plant or algal species (Figure S2). Evidently, spontaneous polymerization is an inherent and widely conserved property of VEL domains.

To identify polymerization-defective mutants of VIN3_VEL_, we performed a semi-systematic mutation screen, generating a series of point mutations in conserved charged residues (as these tend to be surface exposed) and tested their ability to self-associate by SEC-MALS. We thus identified RR>AD, a double mutant that remains predominantly monomeric up to high concentration (Figure 2A). The equivalent double mutation of VEL1_VEL_ (RK>AD) also essentially blocks polymerization (Figure 2B). In a subsequent round of mutation screening based on crystal structures (see below), we identified four additional substitutions of conserved surface-exposed residues of VIN3_VEL_ that strongly attenuated (R541H, R556D, L563D) or blocked (I575D) polymerization (Figure 2C). Importantly, the same point mutations in the corresponding full-length VEL proteins also inhibit their nuclear condensation in plant and mammalian cells (Figures 1 and S1). Therefore, VEL domains behave akin to DIX and SAM domains whose polymerization can be blocked by specific point mutations, which also inhibit dynamic condensation in cells (e.g., Gambetta and Muller, 2014; Schwarz-Romond et al., 2007; Feng et al., 2007).

### Higher-order self-assemblies of VEL domains

**Figure 3:**
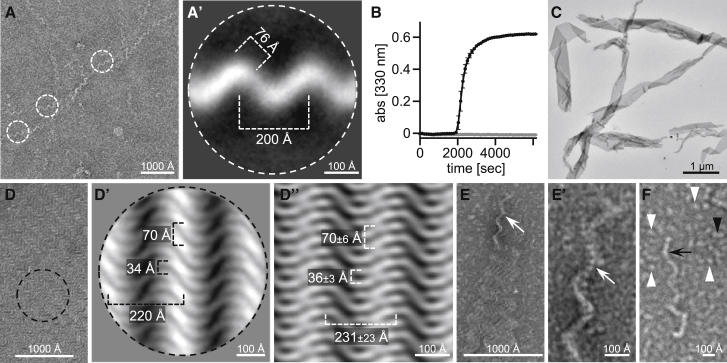
Fiber and lattice superstructures formed by VEL domains (A) EM micrograph of VEL1_VEL_ fiber and corresponding 2D class average (A′), with fiber thickness and pitch indicated. (B) Kinetics of VIN3_VEL_ (residues 500–603) floater formation (black, WT; gray, RR>AD); error bars, SEM (n = 4). (C and D) EM micrographs of VIN3_VEL_ lattices, with dimensions derived from (D′) 2D class averages or (D′′) inverse FFTs of individual lattice sections. (E) VIN3_VEL_ lattice with protruding wavy edge (white arrows), (E′) zoomed in to reveal the beady substructure of apparently intertwined filaments. (F) Magnified views, with individual detached wavy filament composed of beads (black arrow), single round beads (white arrowheads), and two-beaded particles (black arrowheads).

To visualize the VEL polymers observed by SEC-MALS, we examined preparations of purified VEL domains by electron microscopy (EM) after uranyl-acetate staining. VEL1_VEL_ remains soluble to high concentrations (1.5 mM) even after removal of the Lip tag, but over time, this domain begins to form white “floaters” of a silky appearance, visible as superhelical fibrous assemblies in negatively stained EM preparations (Figure 3A). The two-dimensional (2D) class averages of these fibers indicate a width of ∼76 Å (Figure 3A′), i.e., clearly wider than would be expected for a single head-to-tail VEL filament based on its crystal structure (see below), further supporting the notion that these fibers consist of multiple intertwined protofilaments. Importantly, the RK>AD mutant never forms floaters nor fibers. Therefore, the formation of the fibrous superstructures critically depends on polymerization by VEL1_VEL_.

In contrast, VIN3_VEL_ cannot be purified without its Lip tag: as soon as this is cleaved off by TEV protease, VIN3_VEL_ precipitates into floaters. Changes in optical density indicate that their formation plateaus by ∼1 h after the start of TEV cleavage (Figure 3B). As for VEL1_VEL_, VIN3_VEL_ floaters are exclusively observed with the WT domain, whereas RR>AD remains soluble without a tag up to high concentration (2.4 mM; Figure 3B). Uranyl-acetate staining reveals ribbons (Figure 3C), formed from striking lattices (Figure 3D) composed of sideways aligned wavy filaments, with a mean average inter-filament distance of ∼34 Å, as indicated by 2D class averaging (Figure 3D′). Fast Fourier transforms (FFTs) of individual lattice sections (after FFT threshold adjustment to isolate diffraction spots) suggest that the filaments may be aligned in pairs (Figure 3D′′). The same pattern is seen reproducibly in different regions of the grids, indicating that these VIN3_VEL_ lattices are structurally homogeneous and implying a single mode of self-assembly.

Zooming in on the VIN3_VEL_ filaments at the wavy edges of individual lattices (Figure 3E) reveals a beady substructure and suggests intertwined filaments (Figure 3E’). In addition, we observe isolated thin beady filaments, single round beads with a mean average diameter of 32 ± 4 Å (likely free Lip tag), as well as two-beaded particles (putative VEL dimers or uncleaved Lip-VEL; Figure 3F; see also below). Overall, these EM micrographs indicate that VIN3_VEL_ assembles into a rather unusual lattice structure, distinct from the fibrous superstructure of VEL1_VEL_ (Figure 3A) and those previously reported for DIX or SAM domains (Bienz, 2020).

### Head-to-tail polymerization interfaces

**Figure 4:**
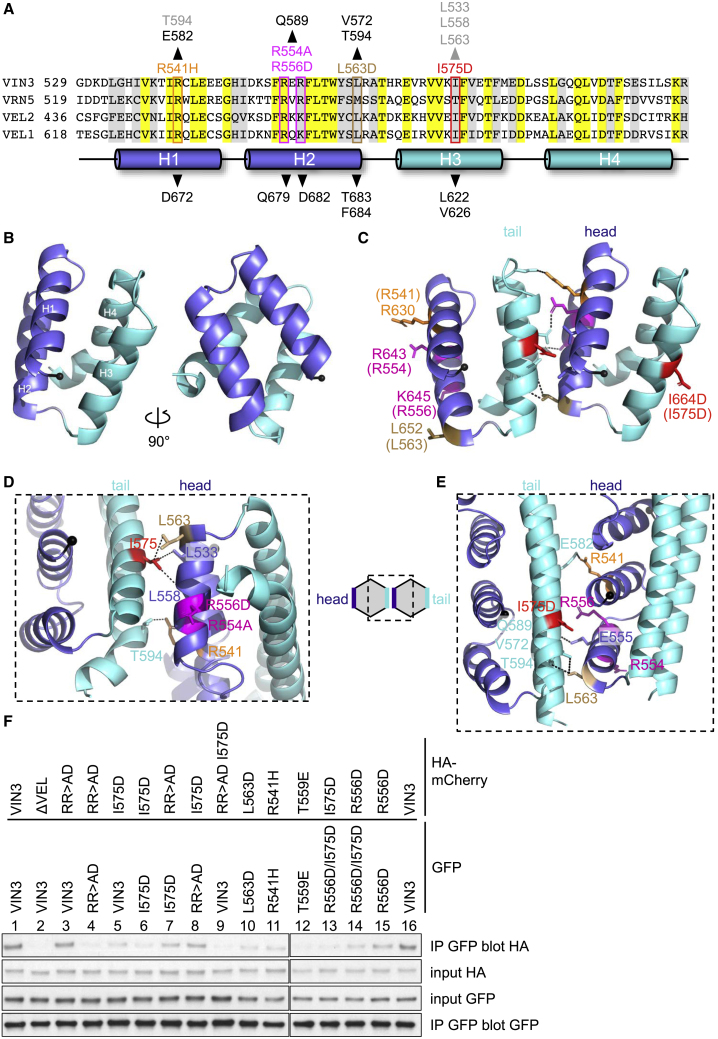
Head and tail surfaces of VEL domains (A) Sequences of VEL domains from *Arabidopsis*, with α-helices indicated underneath; above, VIN3 mutations disabling self-association; arrowheads indicate key interacting residues in VEL1_VEL_ I664D and VIN3_VEL_ I575D (black) or VIN3_VEL_ RR>AD (gray). (B) Ribbon diagrams of the VEL1_VEL_ I664D monomer; blue, head; cyan, tail. (C–E) Ribbon diagrams depicting polymerization interfaces of (C) VEL1_VEL_ I664D, (D) VIN3_VEL_ RR>AD, or (E) VIN3_VEL_ I575D, with residues mediating head-to-tail interactions (dotted lines) in stick (numbering for VIN3_VEL_ in brackets in (C). (F) coIP assays of WT and mutant tagged VIN3 proteins, as in Figure 1I. See also Figure S3 and Table S1.

Next, we sought to determine the crystal structure of VEL domains to identify their self-interacting surfaces. However, we were unable to obtain diffracting crystals for either WT domain because of their pronounced tendency to precipitate into superstructures. Noting that most crystal structures of SAM and DIX domains were determined with polymerization-defective mutants (Bienz, 2020), we switched to this approach and thus succeeded in obtaining diffracting crystals of different mutants (including I664D, I575D, and RR>AD) under diverse conditions (Figure 4A). This allowed us to solve multiple structures for each paralog domain at high resolution (1.84–2.64 Å; Table S1), following labeling with selenomethionine to determine phasing. Importantly, despite being derived from different mutants and crystallographic space groups, individual structures closely resemble each other, with very low root-mean-square deviation (RMSD) values for their backbones (VEL1_VEL_, RMSD: 0.41 Å; VIN3_VEL,_ RMSD: 0.89–1.69 Å), or the backbones of the paralogous domains (RMSD: 1.61–1.77 Å), consistent with the high sequence conservation (Figures 4A and S2).

The VEL domain adopts a compact globular fold, consisting of four α-helices in a quasi-antiparallel “up-down-up-down” configuration, each of which contributes to the hydrophobic core, as illustrated by VEL1_VEL_ (Figure 4B). The same fold is seen for VIN3_VEL_ in solution, as we show below using NMR. The VEL fold is reminiscent of a four-helical bundle (4HB), and the residues whose mutations disable polymerization are typically solvent exposed. Furthermore, these residues form close interactions with residues from a neighboring protomer (often from an adjacent crystal unit cell; Figures 4C–4E), thereby defining one of two complementary surfaces, head or tail, that mediate VEL self-association. As expected, these interacting residues are all invariant or semi-conserved, except for I575, whose counterparts are also hydrophobic in VIN3 paralogs but are typically threonines in VRN5 paralogs (Figures 4A and S2). We used solution-state NMR of a ^13^C-^15^N double-labeled VIN3_VEL_ head mutant (RR>AD) probed with an unlabeled VIN3_VEL_ tail mutant (I575D) to confirm that their unmutated head and tail residues also engage in intermolecular interactions: line broadening of peaks in a ^1^H-^15^N heteronuclear single quantum coherence (HSQC) spectrum of RR>AD upon the addition of I575D was mostly observed on the tail surface (Figure S3), consistent with dimer formation. The same two mutants allowed us to measure an auto-affinity by isothermal calorimetry (ITC) of 1.16 μM (±90 nM) for the head-to-tail interface of VIN3 (Figure S3).

We conclude that the VEL head and tail surfaces are defined largely by three key residues, an isoleucine (VIN3_I575_ or VEL1_I664_) and two basic residues (VIN3_R554,R556_ or VEL1_R643,K__645_), that engage in close hydrophobic or electrostatic interactions, respectively, with partner residues from the apposing complementary surface. These partners tend to be topological equivalents in the different structures except for the isoleucine partners. The latter are located in H2 of VIN3_VEL_, but in H1 of VEL1_VEL_, suggesting an inherent plasticity of these head-to-tail interfaces. These differences affect the orientations of the apposing VEL domains relative to each other. While this could, in principle, explain why the two paralog domains form distinct higher-order assemblies (Figure 3), it is difficult to ascertain this in the absence of determining their structure by cryo-EM. As a further caveat, we also note a “neomorphic” interaction in the RR>AD interface where the R>D substitution allows R556D to interact with R568 at the base of H3 instead of the H4 residues with which R556 interacts in the other structures. Evidently, some caution is needed when extrapolating from the structural details of these interfaces from different mutants to that of the WT domain, although the small RMSDs between the different mutant structures argue that their overall fold is highly similar. We also note that our structures superimpose very closely onto the structures of the WT domains predicted by AlphaFold (Jumper et al., 2021).

Given this slight caveat regarding the RR>AD interface, we sought to validate this interface, designing mutations in other key interacting residues and testing these in cell-based coIP assays in the context of full-length VIN3. As expected, the head mutations L563D, R541H, and T559E and the tail mutation I575D all reduce or abolish self-association without affecting the protein expression levels (Figure 4F). Importantly, individual head and tail mutants retain coIP if tested in *trans* (Figure 4F, lane 8) and so complement each other’s defect, as expected, since they each retain one intact surface for mutual binding. Conversely, if these same mutations are placed in *cis*, this abolishes coIP (Figure 4F, lane 9), again as expected, since neither surface of this mutant is intact. We conclude that VIN3 self-associates in cells through its polymerization interface, as identified by crystallography and confirmed by NMR and coIP experiments in cells. Together with our results from SEC-MALS, this validates the head-to-tail polymerization model for the VEL domain (cartoon in Figure 4) derived from these crystals.

### Assembly of VIN3_VEL_ polymers from domain-swapped dimers

**Figure 5:**
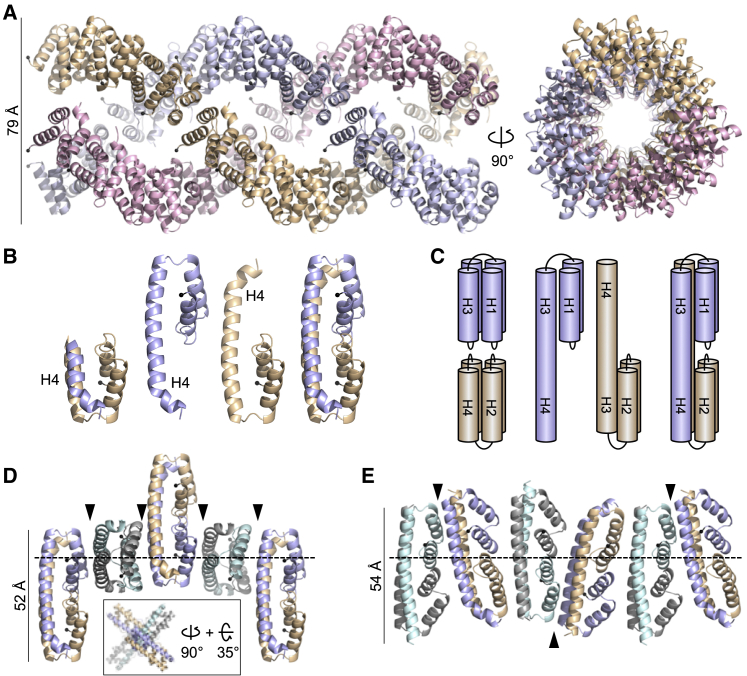
Protofilaments of VEL1_VEL_ and VIN3_VEL_ (A) Ribbon diagram of superhelical fiber assembled from three intertwined VEL1_VEL_ protofilaments (in different colors); right, view from top; black balls, N termini. (B) Ribbon diagrams of two conformations of VIN3_VEL_, with H4 tucked under (left) or extended (middle), and of domain-swapped dimer (right). (C) Cartoon of VIN3_VEL_ monomers and dimer as in (B), illustrating H4 domain swapping. (D and E) Distinct architectures of VIN3_VEL_ protofilaments, as seen in (D) RR>AD (inset, view down polymer) or (E) I575D crystals; arrowheads, polymerization interfaces; dashed lines, planes of polymerization. See also Figure S4.

Next, we looked whether we could identify protofilaments in these VEL crystals. Indeed, VEL1_VEL_ I664D crystals contain right-handed helical filaments composed of 12 protomers per turn that interact through their head and tail surfaces. Three of these filaments intertwine into a superhelical fiber (Figure 5A) whose width (∼79 Å) is similar to that of the WT VEL1_VEL_ fibers observed by EM (Figure 3A), suggesting correspondence between the superhelical fibers observed by these two methods. Neither filaments nor superhelical fibers can be discerned in crystals of RK>AD I664D bearing mutations in both its head and tail surfaces (Figure S4), indicating that the ability of this double mutant to self-assemble is severely compromised even at the ultrahigh protein concentrations reached during crystallization.

VIN3_VEL_ crystals also show protofilaments; however, these are invariably composed of dimers rather than monomers as in VEL1_VEL_. Indeed, dimers are seen in every single crystal, regardless of the mutant, space groups, or crystallization conditions. Interestingly, these dimers result from mutual domain swapping of H4 between individual VIN3_VEL_ monomers whereby molecule A donates its H4 to molecule B in an adjacent VIN3_VEL_ filament, and vice versa (Figure 5B and 5C). This reciprocal exchange preserves the same H4-H3 interactions as seen in the non-swapped globular conformation of VEL1_VEL_ (Figure 4B).

To form filaments, individual VIN3_VEL_ dimers either stack perpendicularly on top of each other in a “cruciform” fashion (in RR>AD; Figure 5D) or they associate pairwise to form tetramers that, in turn, stack pairwise along the polymerization axis (in I575D; Figure 5E). These protofilaments pack into different open-ended superstructures, but without high-resolution structural information from cryoEM, it is impossible to map these onto the lattices of the WT domain observed in negatively stained EM preparations, and so the architecture of the WT VIN3_VEL_ higher-order assemblies remains uncertain. These assemblies may be composed of domain-swapped dimers—apparently a salient feature of VIN3_VEL_, given that domain-swapped dimers are observed in every single crystal, including those from the double-mutant R556D I575D (with mutant head and tail surfaces), even though the latter neither forms filaments nor superstructures (Figure S4). Clearly, VIN3_VEL_, but not VEL1_VEL_, has an intrinsic propensity to undergo domain swapping while forming assemblies during crystallization. It would be challenging to ascertain that this also occurs in solution because the WT VIN3_VEL_ domain is not amenable to analysis in solution, owing to its pronounced tendency to precipitate when untagged.

### A compact globular conformation of the VIN3_VEL_ monomer

**Figure 6:**
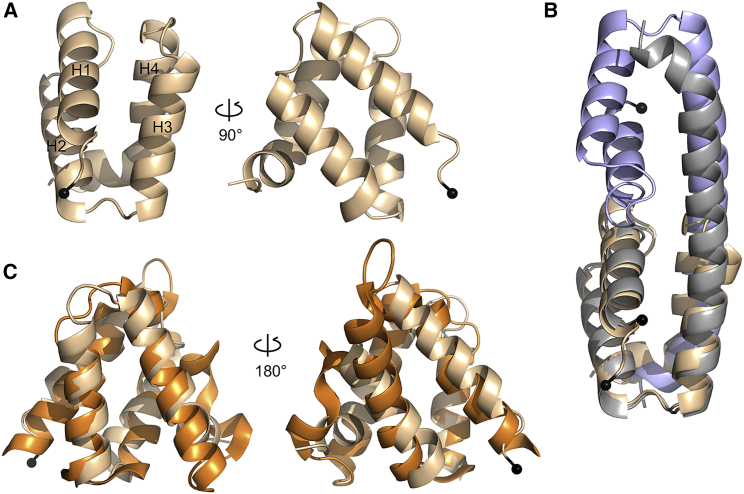
Solution structure of VIN3_VEL_ and comparison with 4HB domains of DNAJ co-chaperones (A) Ribbon diagrams of VIN3_VEL_ RR>AD monomer, reflecting ensemble structure of 10 lowest-energy NMR data-driven CS-Rosetta models, adopting “H4 tucked under” conformation. (B and C) Overlays of RR>AD as in (A) (wheat) with (B) domain-swapped dimer (blue, gray, monomeric subunits in “H4 extended” conformation) as seen in crystals of VIN3_VEL_ RR>AD (PDB: 7O6U) or with (C) 4HB domain of Zuotin (PDB: 4GMQ; orange); backbone RMSD values are (B) 0.46 and (C) 2.92 Å; black balls, N termini. See also Figures S5 and S6.

Because VIN3_VEL_ invariably dimerizes during crystallization, we were unable to determine the structure of the VIN3_VEL_ monomer. To gain insight into its monomeric fold, we used NMR to determine the solution structure of the RR>AD mutant. Complete backbone resonance assignments obtained for a ^13^C-^15^N double-labeled sample of untagged RR>AD as input to the program TALOS-N (Shen and Bax, 2013) allowed us to identify secondary-structure elements and evaluate the degree of disorder along the backbone (Figure S5). This revealed that the helical boundaries in solution coincide with those seen in the VEL1_VEL_ crystal structures. Indeed, the structural modeling by CS-Rosetta (Lange et al., 2012), incorporating backbone chemical shifts plus a limited set of restraints from 50 nuclear Overhauser effects (NOEs) and 72 residual dipolar couplings (RDCs), suggests a compact globular fold of the VIN3_VEL_ monomer in solution, with few violations from the experimental data (Figure 6A): the ensemble of the 10 lowest-energy CS-Rosetta models (from 1,000 candidate structures) converged to a mean backbone RMSD of 0.62 ± 0.08 Å from the lowest for residues 531–599. We conclude that, under these conditions in solution, VIN3_VEL_ adopts a compact globular fold with its H4 tucked under (Figure 6A), like VEL1_VEL_ in the crystal (Figure 4B).

This “H4 tucked under” conformation was confirmed by the random coil index order parameter (RCI-S^2^) values, which indicate a short disordered segment in residues 581–585, suggesting a kink between H3 and H4. The different orientation of H3 and H4 was further confirmed by ^1^*D*_NH_ RDC values measured in Pf1 phage alignment medium (Figure S5). These results reveal that H3 is oriented differently to H4 relative to the major axis of diffusion. Indeed, a comparison between experimental and best-fit ^1^*D*_NH_ RDC values obtained from PALES (Zweckstetter and Bax, 2000) showed a mean correlation coefficient of 0.96 for the CS-Rosetta ensemble. Remarkably, this model superimposes almost perfectly (backbone RMSD 0.46 Å for residues 531–599) onto the domain-swapped VIN3_VEL_ protomers seen within the RR>AD crystals (Figure 6B). Our combined results from NMR and crystallography suggest that VIN3_VEL_ can switch between two conformations, with H4 tucked under when monomeric or extended as in the domain-swapped dimer subunits of the protofilaments seen in the crystals.

### Close VEL domain relatives in universal DNAJ co-chaperones

To identify structural relatives of the globular VEL fold, we conducted DALI searches with the monomeric VEL structures. This resulted in similar lists of highly significant top hits, identifying the 4HB-like domains within yeast Zuotin (Ducett et al., 2013; Leidig et al., 2013) and its mammalian ortholog Zuotin-related factor 1 (ZRF1), also known as DNAJC2 (Shrestha et al., 2019), as the closest structural relatives (Figure 6C; RMSD values: 2.69–3.02 Å, depending on the ortholog and VEL structure). These are followed by the 4HB-like domain of the histone chaperone DAXX (Hoelper et al., 2017) (RMSD values: 3.18–3.29 Å, depending on the VEL structure; Figure S6). Zuotin/ZRF1 are J-proteins belonging to the family of DNAJ co-chaperones, the substrate-targeting subunits of the universal chaperone complexes whose catalytic subunits are the DNAK ATP-dependent unfoldases (Chen et al., 2014; Craig and Marszalek, 2017). DNAK and DNAJ are both relatives of heat-shock proteins (of hsp70 and hsp40, respectively), whereby the latter have homology to α-crystallins that polymerize to form the mammalian eye lens (Ingolia and Craig, 1982). Intriguingly, both DAXX and the nuclear versions of J-proteins also contain histone-binding domains (Elsasser et al., 2012; Shrestha et al., 2019), and ZRF1 functions as a co-factor of the mammalian PcG silencing machinery (Aloia et al., 2015). Of note, the VEL domain and its closest relatives adopt a non-canonical 4HB fold, with atypical angles between individual α-helices, and thus constitute a subclass of the relatively widespread 4HB domain. However, neither ZRF1_4HB_ nor DAXX_4HB_ polymerize, as judged by their SEC-MALS profiles (Figure S6), and so the ability to undergo head-to-tail polymerization represents an exceptional acquisition by the VEL domains within this subclass.

### Polymerization mutations of VIN3 block cold-induced Polycomb silencing of *FLC*

**Figure 7:**
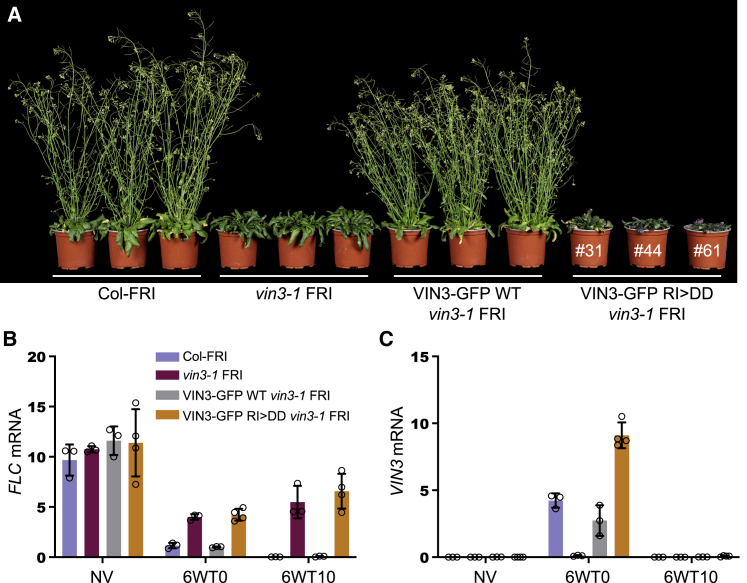
Complementation assays of *vin3* mutant *Arabidopsis* with WT or polymerization-defective VIN3 (A) Representative images of Col-*FRI* WT and *vin3-1* null mutant *Arabidopsis* plants 25 days after cold exposure (6WT25), transformed with WT (single transgene insertions) or RI>DD VIN3-GFP, revealing delayed flowering in RI>DD plants as in untransformed *vin3-1* mutants; numbers indicate individual representative RI>DD lines (see also Figure S7). (B and C) qRT-PCR assays of (B) *FLC* or (C) *VIN3* transcript levels in samples from a single homozygous WT VIN3-GFP transgenic line or from pools of first-generation transgenic lines (n = 36) for VIN3-GFP RI>DD. RNA was extracted before vernalization (non-vernalized [NV]), at the end of a 6-week cold exposure (6WT0), or 10 days after return to warm (6WT10). Data presented are relative to the geometric mean of *UBC* and *PP2A*. Error bars represent standard deviations (n = 3–4 independent leaf pools). See also Figure S7.

To test the function of VEL polymerization in *FLC* silencing in plants, we generated WT and mutant VIN3-GFP transgenes bearing R556D and I575D (RI>DD) for stable transformation of the *Arabidopsis* null mutant *vin3-1 FRI* (Sung and Amasino, 2004). We confirmed by SEC-MALS that the polymerization of this double mutant is completely disabled (Figure S7). We then compared *FLC* expression in stable transgenic lines and untransformed Col-*FRI* control over a vernalization time course before (non-vernalized [NV]), at the end of (6WT0), and 10 days after cold exposure (6WT10). The WT transgene fully rescued the *vin3-1 FRI* vernalization defect (Figures 7A–7C), with *FLC* levels matching the Col-*FRI* control at all stages of the vernalization time course (Figure 7B, compare gray with blue). In contrast, the RI>DD transgene did not complement the *FLC* silencing defect of *vin3-1 FRI,* resulting in derepression of *FLC*, with high *FLC* transcript levels comparable to those seen in the null *vin3-1 FRI* mutant; importantly, the defective *FLC* silencing in the RI>DD rescue lines occurred both during and after cold exposure (Figure 7B, orange). This defect was not due to differences in transgene expression levels as the *VIN3* transcripts accumulated to similar levels in WT and RI>DD lines as in the untransformed Col-*FRI* control (Figure 7C). We also used confocal imaging of lateral root tips to confirm our observations obtained in transiently transfected *N. benthamiana* leaves that the polymerization-disabling mutations in RI>DD neither affect the overall protein levels nor the nuclear accumulation (Figures S1 and S7). We therefore conclude that the failure by this mutant to confer stable *FLC* silencing in transgenic *vin3-1 FRI* plants is likely to be the consequence of its failure to polymerize, which indicates the physiological relevance of our findings based on biochemical *in vitro* assays of recombinant VEL domains.

## Discussion

By studying *Arabidopsis* VEL proteins that engage in heritable PcG silencing during vernalization, we found that their purified VEL domains polymerize spontaneously into short filaments and striking fibrous or latticed superstructures. Our evidence, based on polymerization-defective mutants, indicates that this property enables VEL proteins to assemble dynamic molecular condensates in plant cells, like those assembled by Polycomb SAM domain proteins in animal cells (Isono et al., 2013). The VEL domain adopts a 4HB-like fold related to one found in ancient DNA co-chaperones. Therefore, this fold is structurally distinct from those of DIX and SAM, the only other domains known to undergo spontaneous dynamic head-to-tail polymerization (Bienz, 2020) to mediate assembly of biomolecular condensates (Banani et al., 2017).

Our evidence implicates biomolecular condensation as an important mechanism in PRC2 silencing at *FLC*. The components of biomolecular condensates transition reversibly between diffuse and locally condensed pools, attaining a high local concentration of ligand binding sites when condensed (“emergent multivalency”), which imparts a high binding avidity for their effectors or substrates (Abbondanzieri and Meyer, 2019; Banani et al., 2017; Banjade and Rosen, 2014; Bienz, 2014). This mechanism could therefore provide a multivalent assembly platform with high avidity for effectors, for example, previously identified co-repressors such as HDA19, ASAP, or TOPLESS (Collins et al., 2019; Questa et al., 2016). Furthermore, polymerization-dependent avidity could facilitate PRC2 association with *FLC* and promote the anchoring and spreading of PcG complexes at this locus, which further consolidates the silencing process (Yang et al., 2017). Therefore, VEL-dependent biomolecular condensation could provide the positive feedback necessary to maintain inheritance of the silencing complex to both daughter DNA strands at replication. It could thus be the mechanism invoked by Lövkvist et al. (2021) for persistence of memory at the *FLC* locus.

The vast majority of DIX and SAM condensates assemble from filaments composed of monomeric subunits (Bienz, 2014, 2020). The same is true for VEL1 filaments, whereas the VIN3 filaments in the crystals are composed of dimeric VIN3_VEL_ subunits (Figure 5B). Interestingly, VIN3_VEL_ dimerizes by domain swapping, a process that typically involves overcoming a high energetic hurdle that separates monomer from dimer, with slow interconversion rates between the two (Rousseau et al., 2003). Dimerization by domain swapping can therefore be switch-like, which in the case of VIN3_VEL_ might explain why this domain self-assembles precipitously into lattice superstructures (Figures 3B–3F), in contrast to VEL1_VEL_, which is far less precipitous. This unusual property of VIN3_VEL_ may assist VIN3 in its role of initiating PcG silencing at *FLC* (Yang et al., 2017): it is plausible that VIN3 is monomeric when its expression is first induced by a cold spell, but the progressive increase of its nuclear concentration in cold-exposed cells (Greb et al., 2007; Sung et al., 2006) might allow it to dimerize by domain swapping and polymerize into larger assemblies, in part explaining the stochastic switch to silencing at the *FLC* locus in response to external cues (Whittaker and Dean, 2017).

The Polycomb group of genes were discovered in *Drosophila* through genetic analysis (Jurgens, 1985). Extensive subsequent work provided key insights into the basic functions of the two main Polycomb complexes, PRC1 and PRC2 (Simon and Kingston, 2009). In addition, the *Drosophila* Polycomb system also includes a third Polycomb complex called the Pleiohomeotic Repressive Complex (PhoRC), whose Pho subunit binds directly to DNA, thereby tethering PRC1 and PRC2 to Polycomb target genes (Frey et al., 2016). Together, PRC1 and PhoRC complexes contain three distinct SAM proteins whose homo- and heteropolymerization are critical for this tethering process (Frey et al., 2016; Alfieri et al., 2013; Kang et al., 2015). Polymerizing SAM proteins undergoing equivalent interactions with their Polycomb partners have also been found in other animals including mammals (Chittock et al., 2017; Alfieri et al., 2013). Furthermore, it has been shown for one of these SAM proteins (called Phc2) that its ability to confer dynamic clustering of PRC1 through SAM-dependent polymerization is critical for stable Polycomb silencing in murine cells (Isono et al., 2013). Notably, SAM-containing Polycomb proteins have not been found in plants (Huang et al., 2019) whose Polycomb systems are rather divergent from that in animals, with PRC2 being the most conserved across the two kingdoms (Bieluszewski et al., 2021).

Based on our study of the VEL domain and its function during vernalization, we envisage that the head-to-tail polymerizing activity of VEL proteins in *Arabidopsis* is equivalent to that conferred by the SAM Polycomb proteins in animals. For example, polymerization may serve to facilitate efficient capture or retention of Polycomb complexes at chromatin target sites or clustering between these sites in the nucleus. However, whereas the primary target for the polymerizing activity of SAM proteins in animals is clearly PRC1, available evidence suggests that VEL-dependent polymerization assists primarily PRC2 in plants. Future studies are necessary to pinpoint the step(s) during the transcriptional silencing of the *FLC* locus facilitated by VEL-dependent polymerization and, indeed, to what extent this can be generalized to other Polycomb silencing events in plants, outside vernalization. Whatever the case, our complementation tests (Figures 7 and S7) suggest that, similarly to the SAM-dependent polymerization of Polycomb proteins in animals, the VEL-dependent polymerization of VEL proteins underpins the ability of plant Polycomb complexes to promote heritable long-term transcriptional silencing.

In conclusion, our finding of a polymerizing domain in VEL proteins suggest that the Polycomb system has co-opted fundamentally the same mechanistic principle in animals and plants—namely the use of domains capable of homo- or hetero-polymerization through their head and tail surfaces—to confer stable tethering of silencing complexes at target loci. The salient features of these domains are their ability to assemble multivalent nuclear condensates with high avidity for effectors and their dynamic behavior allowing rapid remodeling of complexes, which renders them eminently suitable for transcriptional silencing in response to external cues. Our evidence suggests that the memory of winter relies on self-templated reversible VEL polymerization.

### Limitations of the study

The limitations of our study include our inability to determine the crystal structure of WT VEL domains or to study their behavior in solution without a tag, owing to their pronounced tendency to polymerize spontaneously. Another shortcoming is that we have not yet fully exploited our *Arabidopsis* transgenic plants to analyze in depth how defective polymerization affects Polycomb-induced changes at *FLC.* Future genome-wide studies are also required to assess to what extent polymerization-defective VIN3 affects the Polycomb-mediated silencing of loci beyond *FLC* and to define the specific steps during assembly or function of Polycomb silencing complexes that rely on VEL-dependent polymerization.

## STAR★Methods

### Key resources table

**Table:** 

REAGENT or RESOURCE	SOURCE	IDENTIFIER
**Antibodies**		

α-GFP (rabbit)	Sigma-Aldrich	Cat#G1544
α-Flag (mouse)	Sigma-Aldrich	Cat#F1804
α-Flag (rabbit)	Sigma-Aldrich	Cat#F7425
α-HA (rat)	Sigma	Cat#3F10
α-HA (rabbit)	Abcam	Cat#ab9110
α-β-tubulin (mouse)	Sigma	Cat#T4026
HRP conjugated Goat α-Rabbit	Santa Cruz Biotechnology	Cat#sc-2301
HRP conjugated Goat α-Mouse	Santa Cruz Biotechnology	Cat#sc-2005
Alexa Fluor 488 conjugated Goat α-Rabbit	Life Technologies	Cat#A11008
Alexa Fluor 546 conjugated Goat α-Mouse	Life Technologies	Cat#A11003

**Chemicals, peptides and recombinant proteins**		

Ni-NTA Agarose	Qiagen	Cat#30210
α-FLAG M2 Affinity Gel	Sigma	Cat#A2220
GFP-TRAP	Chromotek	Cat#gta-20
Polyethylenimine, linear, MW25000	Polysciences	Cat#23966
Lipofectamine2000	Invitrogen	Cat#11668019
FugeneHD	Promega	Cat#E2311
EDTA-free Protease Inhibitor Cocktail	Roche	Cat#04693159001
VectaShield with DAPI	Vector Laboratories	Cat#H-1200
Isopropyl-β-D-1thiogalactopyranoside, IPTG	Sigma-Aldrich	Cat#I6758
Imidazole	Sigma-Aldrich	Cat#56748
KOD DNA polymerase	Merck Millipore	Cat#71086-4
DNAse I	Sigma-Aldrich	Cat#D4527
ECL Western Blotting Detection Reagent	Amersham	Cat#RPN2106
Skimmed milk powder	Marvel	N/A
Sarkosyl	BDH Chemicals	Cat#44275
NP-40	AppliChem	Cat#A1694
InstantBlue Coomassie Protein Stain	Abcam	Cat#ab119211
Iodoacetamide Bio-Ultra	Sigma-Aldrich	Cat#I1149
Poly-L-Lysine Hydrobromide	Sigma-Aldrich	Cat#P1399
Opti-MEM medium	Gibco	Cat#31985-070
DMEM + Glutamax	Gibco	Cat#31966-021
Perfluoropolyether cryo oil	Hampton Research	Cat#HR2-814
Uranyl Acetate EM Solution 2%	TAAB	Cat#U001/S/2/25
Silwet L-77	BHGS	Cat#SILXXX000001
TURBO DNase	Invitrogen	Cat#AM2239
SuperScript IV Reverse Transcriptase	Invitrogen	Cat#18090050
LightCycler 480 SYBR Green I Master	Roche	Cat#04887352001
RNaseOUT Recombinant Ribonuclease Inhibitor	Invitrogen	Cat#10777019
UVette	Eppendorf	Cat#0030106300
Glass coverslips 22 × 22 mm	VWR	Cat#631-0124
μ-Slide 8 well	Ibidi	Cat#80827
300-mesh carbon film on copper EM grids	EMR	Cat#C300Cu100
Whatman No. 1 blotting paper	GE Healthcare Life Sciences	Cat#WHA10010155
100mm style cell culture dish, TC-treated polystyrene, 89 mm × 19 mm	Corning	Cat#353003

**Critical commercial assays**		

HiPure Plasmid Miniprep Kit	Invitrogen	Cat#K210011
HiPure Plasmid Midiprep Kit	Invitrogen	Cat#K210005
Gel extraction kit	Qiagen	Cat#28704

**Deposited data**		

VEL1 I664D	This paper	www.rcsb.org/7O6W
VEL1 R643A K645D I664D	This paper	www.rcsb.org/7O6V
VIN3 I575D	This paper	www.rcsb.org/7OQV
VIN3 R554A R556D	This paper	www.rcsb.org/7O6U
VIN3 R556D I575D	This paper	www.rcsb.org/7O6T
VIN3 RR>AD, NMR model	This paper	https://bmrb.io/50800

**Experimental models: Cell lines**		

HEK293T	ATCC	Cat#CRL-3216
COS-7	ATCC	Cat#CRL-1651
HeLa	ATCC	Cat#CCL-2

**Experimental models: Organisms/strains**		

*Arabidopsis thaliana* accession Col-*FRI*^*SF2*^	Standard accession	N/A
*A. thaliana vin3-1 FRI*	Sung and Amasino, 2004	N/A
*A. thaliana pVIN3::VIN3-GFP vin3-1 FRI*	This paper	N/A
*A. thaliana pVIN3::VIN3-GFP R556D I575D vin3-1 FRI*	This paper	N/A
*Agrobacterium tumefaciens GV3101 C58(pMP90)*	Intact Genomics	Cat# 1282-12

**Recombinant DNA**		

Plasmid: pENTR pVIN3::VIN3-GFP	Questa et al., 2016	N/A
Plasmid: pENTR: pVIN3::VIN3-GFP R556D I575D	This paper	N/A
Plasmid: pEGFP-C1-VIN3 FL	This paper	N/A
Plasmid: pEGFP-C1-VEL1 FL	This paper	N/A
Plasmid: pEGFP-C1-VRN5 FL	This paper	N/A
Plasmid: pEGFP-C1-HA-VIN3 FL	This paper	N/A
Plasmid: pmCherry-HA-VIN3 FL	This paper	N/A
Plasmid: pmCherry-HA-VEL1 FL	This paper	N/A
Plasmid: pmCherry-HA-VRN5 FL	This paper	N/A
Plasmid: pFlag-dsRED-VEL1 FL	This paper	N/A
Plasmid: pFlag-dsRED-VIN3 FL	This paper	N/A
Plasmid: pLipK-VIN3 VEL	This paper	N/A
Plasmid: pLipK-VEL1 VEL	This paper	N/A
Plasmid: pLipK-VRN5 VEL	This paper	N/A
Plasmid: p6xHis-VIN3 VEL	This paper	N/A
Plasmid: p6xHis-VEL1 VEL	This paper	N/A
Plasmid: p6xHis-VRN5 VEL	This paper	N/A
Plasmid: pLipK-Phoenix VEL	This paper	N/A
Plasmid: pLipK-Zostera VEL	This paper	N/A
Plasmid: pLipK-Amborella VEL	This paper	N/A
Plasmid: pLipK-Pinus VEL	This paper	N/A
Plasmid: pLipK-Picea VEL	This paper	N/A
Plasmid: pLipK-Gnetum VEL	This paper	N/A
Plasmid: pLipK-Ginkgo VEL	This paper	N/A
Plasmid: pLipK-Cycas VEL	This paper	N/A
Plasmid: pLipK-Equisetum VEL	This paper	N/A
Plasmid: pLipK-Selaginella VEL	This paper	N/A
Plasmid: pLipK-Treubia VEL	This paper	N/A
Plasmid: pLipK-Sphagnum VEL	This paper	N/A
Plasmid: pLipK-Spirogloea VEL	This paper	N/A
Plasmid: pLipK-Chara VEL	This paper	N/A
Plasmid: pCAMBIA 1300 p35S: Ω-GFP-VIN3	This paper	N/A
Plasmid: pCAMBIA 1300 p35S: Ω-GFP-VIN3 R554A R556D	This paper	N/A
Plasmid: pCAMBIA 1300 p35S: Ω-GFP-VIN3 I575D	This paper	N/A
Plasmid: pSLJ-DEST (based on pSLJ755I6)	Jones et al., 1992	N/A
Plasmid: pSLJ-VIN3::VIN3-GFP	This paper	N/A
Plasmid: pSLJ-VIN3::VIN3-GFP R556D I575D	This paper	N/A

**Software and algorithms**		

MacVector v17.0.5	MacVector Inc	https://macvector.com
Prism v8.4	GraphPad	https://www.graphpad.com
PyMOL version 2.1	Schrödinger	N/A
CCP4i suite V7.0.078	Evans and Murshudov, 2013	CCP4
ITC200, Malvern, version 1.30.0	Malvern Sciences	N/A
MicroCal PEAQ-ITC Analysis, version 1.1.0.1262	Malvern Sciences	N/A

**Other**		

OneKP consortium database	Matasci et al., 2014	www.onekp.com
Phytozome ver11	Joint Genome Institute (USA)	https://phytozome-next.jgi.doe.gov/
JACKHMMER v2.41.1	EMBL-EBI Potter et al., 2018	https://www.ebi.ac.uk/Tools/hmmer/search/jackhmmer

### Resource availability

#### Lead contact

Requests for further information or reagents should be directed to the lead contact, Mariann Bienz (mb2@mrc-lmb.cam.ac.uk). Specific requests regarding plant materials should be directed to the co-corresponding author Caroline Dean (caroline.dean@jic.ac.uk).

#### Materials availability

There are no restrictions on the availability of materials and reagents mentioned in this work.

### Experimental model and subject details

#### Plant models: *Arabidopsis thaliana*; *Nicotiana benthamiana*

Arabidopsis plants were cultivated in soil in a glasshouse with controlled 22°C 16 h day and 20°C 8 h night conditions before vernalization (non-vernalized, NV) and after vernalization. Plants were vernalized at constant 5°C with 8 h day and 16 h night conditions in a controlled environment growth chamber. Nicotiana plants were cultivated in soil in a glasshouse under the same conditions as the Arabidopsis plants.

#### Cell cultures

HEK293T, HeLa and COS-7 cells were cultured on glass coverslips in 6-well culture dishes in DMEM (Gibco), supplemented with 10% fetal bovine serum (FBS) and penicillin/streptomycin at 37°C in a humidified atmosphere with 5% CO_2_, and regularly screened for mycoplasma.

### Method details

#### Generation of plasmids

VEL sequences (VIN3, Q9FIE3; VEL1, Q9SUM4; VRN5, Q9LHF5) for *in vitro* and cell-based assays were amplified by polymerase chain reaction (PCR) from either plasmid templates (Greb et al., 2007) or synthetic genes (gBlocks, IDT), cloned into mammalian or bacterial expression vectors (for designations, see key resources table) by restriction-free cloning. For VEL1_VEL_ structures, p6xHis-VEL1_VEL_ I664D or p6xHis-VEL1_VEL_ I664D R643A K645D (residues 616–692) from *Arabidopsis thaliana* VEL1 were used. For VIN3_VEL_ structures, p6xHis-VIN3_VEL_ R556D I575D or p6xHis-VIN3_VEL_ R554A R556D (residues 529–603) from *Arabidopsis thaliana* VIN3 were used. For *in vitro* assays and EM, bacterial expression vectors pLipK-VIN3_VEL_ (residues 529–603, unless specified otherwise) and pLipK-VEL1_VEL_ (residues 589–692) were used. Point mutations and deletions were generated by Quikchange, using KOD DNA polymerase (Merck Millipore). All plasmids were verified by sequencing.

#### Phylogenetic analysis

Protein sequences of VEL orthologs were from Phytozome ver11 (https://phytozome-next.jgi.doe.gov/) or from the OneKP consortium (www.onekp.com) (Matasci et al., 2014), or retrieved from JACKHMMER (Potter et al., 2018).

(https://www.ebi.ac.uk/Tools/hmmer/search/jackhmmer). Alignments of protein sequences were done with MacVector (MacVector Inc) using the ClustalW algorithm.

#### Protein expression and purification

6xHisLip- or 6xHis-tagged recombinant proteins were purified from BL21(DE3) pRARE2 *E. coli* bacterial strains. Bacteria were grown in LB media supplemented with appropriate antibiotic to OD_600_ 0.6, then dropped to a lower temperature (16–24°C) and induced at OD_600_ 0.8 by addition of 0.4 mM isopropyl β-D-1-thiogalactopyranoside (IPTG). Proteins were expressed for 3 h or overnight. Bacteria were harvested by centrifugation, cell pellets shock-frozen in liquid nitrogen and stored at −80°C until use. Cell pellets were resuspended in lysis buffer (25 mM Tris-HCl pH 8, 200 mM NaCl, 20 mM imidazole, 10 μg/mL DNAse, EDTA-free protease inhibitor cocktail) and lysed either by high-pressure homogenization with an Emulsiflex C-3 (Avestin) or sonication (Branson). Lysates were cleared by ultracentrifugation (140,000× *g*, 30 min, 4°C) and mixed with Ni-NTA agarose. Beads were washed multiple times with lysis buffer, and 6xHis-tagged protein was eluted with lysis buffer supplemented with 500 mM imidazole. Each protein was purified by SEC, and protein purity was assessed by SDS-PAGE. Selenomethionine-labeled samples were expressed in M9 minimal medium supplemented with 0.4% glucose, antibiotics, trace elements and 30 mL overnight culture per liter expression culture. Cultures were grown at 37°C to OD_600_ 0.6, at which point individual amino acids (0.4 g/L lysine, threonine, phenylalanine and 0.2 g/L leucine, isoleucine, valine and selenomethionine) were added. Cells were induced at OD_600_ 0.8 with IPTG and processed essentially as described above.

#### Protein crystallization and data collection

6xHis-TEV-VIN3_VEL_ and 6xHis-TEV-VEL1_VEL_ bearing polymerization-deficient point mutations were cleaved by TEV protease (TEV:protein ratio 1:80) overnight at 4°C. Cleaved tags were removed by running the cleaved protein on a HiLoad 26/600 Superdex 75 pg column (GE Healthcare) in 25 mM Tris pH 7.4, 200 mM NaCl, 1 mM DTT and 0.06% NaN_3_. Pure fractions of VEL proteins were concentrated with a 3 kD MWCO Vivaspin 20 concentrator (Sartorius) to 4–25 mg/mL. Prior to crystallization, 1 mM TCEP was added, and samples were cleared by centrifugation for 15 min at 100,000 rcf. Crystallization trials with multiple commercial crystallization kits were performed in 96-well sitting-drop vapor diffusion plates (Molecular Dimensions) at 18°C and set up with a mosquito HTS robot (TTP Labtech). Drop ratios of 0.2 μL + 0.2 μL (protein solution + reservoir solution) were used for coarse and fine screening. Initial hits were obtained under multiple conditions and optimized subsequently. Data were collected from crystals harvested from following conditions: VEL1 I664D (7O6W), 0.25 M (NH_4_)H_2_PO_4_, glycerol as cryo protectant; VEL1 R643A K645D I664D (7O6V), 18% w/v PEG 3350, 0.2 M sodium acetate trihydrate, glycerol as cryo-protectant; VIN3 I575D (7OQV), 1 M potassium phosphate monobasic, 3% v/v 2-propanol, 0.1 M sodium cacodylate pH 6.5, glycerol as cryo protectant;

VIN3 R554A R556D (PDB: 7O6U), 15% v/v ethanol, 0.1 M citrate pH 5.5, 0.2 M lithium sulfate, perfluoropolyether cryo oil (Hampton Research) for harvesting crystals; VIN3 R556D I575D (PDB: 7O6T), 10% w/v PEG 8K, 20% v/v ethylene glycol, 0.06 M Morpheus Divalents, 0.1 M Morpheus Buffer System 2 pH 7.5.

To ensure cryo-protection, crystal-containing drops were either mixed with 25% glycerol in reservoir solution, or perfluoropolyether cryo oil was used prior to picking and flash freezing in liquid nitrogen. Diffraction data were collected at the Diamond Light Source (DLS, UK) on beamlines I24 and I04. For data collection, wavelengths optimal for selenomethionine were used.

Data processing was performed with XIA2 DIALS and scaled using Aimless from CCP4 (Collaborative Computational Project, Number 4, 1994) (Evans and Murshudov, 2013). Each structure was solved by single-wavelength anomalous dispersion (SAD) technique using Crank-2 from CCP4 suite of programs. Structure refinement was performed with REFMAC followed by manual examination and rebuilding of the refined coordinates in the program COOT (Emsley et al., 2010). Color figures were prepared with PyMOL (Schrödinger). Calculation of backbone RMSD for the various VEL structures was done with TM-align (https://zhanglab.ccmb.med.umich.edu/TM-align/). Searches for related VEL folds were performed with DALI (http://ekhidna2.biocenter.helsinki.fi/dali/).

#### NMR

^13^C-^15^N double-labeled VEL domains bearing polymerization-deficient point mutations were analyzed in 25 mM phosphate pH 6.7, 150 mM NaCl buffer, 5% v/v D_2_O. Spectra were recorded using Bruker Avance III spectrometers operating at 600 or 800 MHz ^1^H frequency, with 5 mm inverse-detect cryogenic probes and a sample temperature of 283 K, and using unmodified Bruker pulse programs. Backbone resonance assignments were obtained for a ^13^C-^15^N double-labeled sample of 300 μM VIN3_VEL_ RR>AD from fast-HSQC and 3D HNCO, HN(CA)CO (0.05 ppm/point C′), CBCA(CO)NH and HNCACB (0.25 ppm/point C_aliphatic_). Assignments of Hα and all methyl groups were obtained from 2D {^1^H,^13^C}-HSQC and 3D H(C)CH- and (H)CCH-TOCSY spectra with 12 ms DIPSI-2 spin-lock. Leucine and valine methyl signals were assigned stereospecifically from a {^1^H,^13^C}-constant-time-HSQC obtained from a separate sample grown on 10% U-^13^C, 90% unlabeled glucose. A partial assignment of Phe and Val aromatic ^1^H resonances was obtained from 2D (HB)CB(CGCD)HD and (HB)CB(CGCDCE)HE spectra. ^13^C-edited ^1^H–^1^H NOESY (800 MHz) was recorded with 100 ms NOE mixing time. ^1^*D*_NH_ residual dipolar couplings (RDC) were measured from the difference in ^15^N dimension splitting in IPAP-HSQC spectra (0.25 Hz/point digital resolution ^15^N) recorded in the presence or absence of 12 mg/mL Pf1 phage (Asla Biotech). The interaction between 300 μM ^13^C-^15^N VEL RR>AD and 100 μM VEL I575D was monitored by comparison of peak heights in ^1^H-^15^N fast-HSQC spectra (M. F. & T. J. R., unpublished). Frequencies were referenced according to the unified scale, with the ^1^H signal of internal 150 μM dimethylsilapentane sulfonate (DSS) at 0.0 ppm. All spectra were processed with TopSpin version 3.2 (Bruker) and analyzed using NMRFAM-Sparky version 1.3 (Lee et al., 2015).

Complete assignments for H_N_, Hα, N, Cα, Cβ and C′ resonances of VIN3_VEL_ RR>AD (529-603) were input to TALOS-N (online server) (Shen and Bax, 2013) and the CS-Rosetta server (Lange et al., 2012), to generate 3000 structural fragments. The fragment files were then used with CS-Rosetta version 3.8, adding experimental restraints for 50 ^1^H–^1^H NOE identified for resolved signals from methyl groups and 72 ^1^*D*_NH_ RDC. RDC for disordered backbone amides, as judged by TALOS random coil index order parameters (RCI-S^2^) <0.8, were excluded as restraints. All NOE restraints were applied with the same boundaries (lower 1.8 Å, upper 5.0 Å). Structure statistics were compiled for an ensemble of the 10 lowest energy structures from 1000 calculated.

#### SEC-MALS

Purified recombinant proteins were quantified by NanoDrop using the protein-specific extinction coefficient and diluted to the desired concentration (2 mg/mL unless stated otherwise). SEC-MALS was performed in PBS with 1 mM DTT on a Superdex200 10/300 GL column (GE Healthcare) using an Agilent 1200 Series chromatography system coupled to a DAWN Heleos II multi-angle light scattering detector as well as an Optilab rEX refractive index detector (Wyatt Technology). 100 μL sample was used per run at a flow rate of 0.5 mL/min. BSA was used for calibration. Baseline correction, selection of peaks and calculation of molecular masses was performed with the Astra 6.1 software package.

#### ITC

To determine the affinity between VIN3_VEL_ monomers, ITC was carried out at 25°C with an iTC 200 Microcalorimeter (GE Healthcare). Titrations consisted of 19 consecutive 2 μL injections of 1 mM 6xHisLip-VIN3_VEL_ I575D (following a pre-injection of 0.5 μL) into 100 μM 6xHisLip-VIN3_VEL_ RR>AD at time intervals of 180s with constant stirring at 750 rpm, in 25 mM Tris pH 7.4, 200 mM NaCl, 0.5 mM DTT and 0.06% NaN_3,_ and the data were analyzed using MicroCal PEAQ-ITC Analysis Software (1.1.0.1262, Malvern Sciences).

#### VEL floater assay

Purified 6xHisLip-tagged VIN3_VEL_ (residues 500–603) was diluted to a final concentration of 15 mg/mL in a 100 μL reaction volume in a clear bottom 96-well plate (Nunc). TEV protease and DTT were mixed bubble-free in reaction buffer (200 mM NaCl, 25 mM Tris pH7.4, 0.06% NaN_3_, 5 mM DTT and TEV protease in a 1:50 ratio, TEV:protein). Measurements were done in a Pherastar (BMG Labtech) at 30°C and 330 nm (20 flashes per cycle) in 2 min intervals with 20 s shaking (250 rpm, 3 mm double orbital) prior to measurement. Values were subtracted against a blank well and analyzed in Prism8 (Graphpad). Experiments were performed four times from which SEM values were calculated.

#### Negative staining for EM analysis

For EM analysis of purified VIN3_VEL_ or VEL1_VEL_ by negative staining, 300-mesh carbon film on copper EM support grids (C300Cu100, Electron Microscopy Sciences, Pennsylvania, USA) were glow-discharged (30 s; 35 mA; ∼0.23 mbar) using an Edwards Sputter Coater S150B (Edwards High Vacuum Products, Crawley, UK). 4 μL of purified VIN3_VEL_ (∼35 μM) or VEL1_VEL_ (∼138 μM) was applied to the freshly glow-discharged EM support grids and incubated for 1 min at room temperature. Excess sample was removed with No. 1 Whatman (GE Healthcare Life Sciences, England) blotting paper, 20 μL 2% uranyl acetate (TAAB Laboratories Equipment Ltd, UK) was applied to the grid and immediately blotted away before adding fresh 20 μL 2% uranyl acetate for 45 s at room temperature. Excess stain was blotted with Whatman blotting paper, and the grids were air dried. Negatively stained grids were mounted on a single tilt side-entry room temperature specimen holder (Thermo Fisher Scientific, USA) and transferred to either a 200 keV Tecnai F20 (Thermo Fisher Scientific, USA) or a 120 keV Tecnai G2 Spirit Twin (Thermo Fisher Scientific, USA) transmission EM. Electron micrographs were collected at a 26k, 50k or 62k magnification with calibrated image pixel sizes of 3.95, 2.09 and 1.64 Å/pixel, respectively, and were recorded in TIFF format on a Falcon III (Thermo Fisher Scientific, USA) direct electron detector or Orius CCD camera (Ultrascan 1000XP, Gatan, USA). For 2D structural analysis, the grids were transferred to a Tecnai F20 transmission EM, and images were recorded automatically on a Falcon III direct electron detector (Thermo Fisher Scientific, USA) operating in linear mode using EPU software (v1.11.1, Thermo Fisher Scientific, USA). The exposure time was set to 2 s, which provided an accumulated dose of 60 electrons per Å^2^. The images were recorded at a defocus of - 2.2 μm, and an objective aperture of 100 μm diameter was inserted during data acquisition.

#### EM image processing

Electron micrographs were imported to Relion 3.1.1 (Zivanov et al., 2018) and contrast transfer functions (CTFs) of the micrographs were estimated using CTFFIND4 (Rohou and Grigorieff, 2015). VIN3_VEL_ fibers from 68 selected micrographs were manually picked using Relion’s start-end coordinate parameter by setting the particle diameter to 160 Å. To obtain two-dimensional information of VIN3_VEL_ on a multi-fiber scale, a total number of 4933 particles were extracted with 880 × 880 pixel box and classified into 15 classes using reference-free 2D-classification inside Relion. For two-dimensional structural analysis of VIN3_VEL_ on a single-fiber scale, a total number of 32,577 particles were extracted with a 260 × 260 pixel box and classified into 5 classes. Of the entire dataset, 43 micrographs (where self-folding of VIN3_VEL_ filament-sheets could be appreciated) were selected, and particles were manually picked using Relion’s start-end coordinate parameter. A total number of 913 particles with a 260 × 260 pixel box were extracted in this case, and classified into 10 classes using reference-free 2D-classification inside Relion.

VEL1_VEL_ fibers from 127 selected micrographs were manually picked using Relion’s start-to-end parameter by setting the particle diameter to 150 Å. A total number of 3607 particles were extracted with a 240 × 240 pixel box and classified into 25 classes using reference-free 2D-classification inside Relion. To analyze the structure of the VIN3_VEL_ lattice, ImageJ (v 1.53e; Rasband, 1997–2018) was used to create FFTs of selected micrograph areas. Low pass filters were applied to the FFTs using the threshold adjustment tool, with minimum threshold values set at ≥164, sufficient to select only the diffraction maxima. Diffraction pattern maxima fell into three distinct orders, which were each singled out in turn, by deleting the other two. Inverse FFTs were then generated. In these images, brightness/contrast was auto-adjusted in ImageJ, and intensity profiles were plotted along the main axis of periodicity and distances between peaks were measured. 180–300 measurements were taken from inverse FFTs of five separate VIN3_VEL_ lattices. The mean average ±standard deviation was calculated in Excel (Microsoft Office Professional Plus, 2019).

#### Immunofluorescence of mammalian cells

HEK293T, HeLa and COS-7 cells were transfected with 1 μg total DNA and 3.5x PEI (HEK293T and HeLa cells), or FugeneHD (COS-7 cells). For GFP-VIN3, 1 μg of plasmid was expressed for 24 h, whereas for GFP-VEL1, 200 ng plasmid was topped up with 800 ng of pCMV-tag2b empty vector and expressed for 17 h. PBS washed cells were fixed on coverslips with 4% formaldehyde in PBS for 20 min and subsequently permeabilized by 0.5% Triton X-100 in PBS for two minutes. Coverslips were washed with PBS-T and embedded with VectaShield with DAPI mounting media. Images were acquired with identical settings using a Zeiss 710 Confocal Microscope using ‘best signal’ setting (Smart Setup, ZEN software, Zeiss). For quantitation of protein distribution, >100 cells of each transfected construct were classified into three groups (diffuse, grainy and punctate). Within the field of view, only cells with intact nucleus (as judged by DAPI staining) were considered.

#### FRAP analysis

HeLa cells were grown in 8-well μ-Slide chambers (Idibi). Chambers were pre-treated with 50 μg/mL poly-L-lysine (Sigma), and cells were seeded at approximately 50% confluency. After attachment, cells were transfected with 100 ng total DNA with 3.5x Lipofectamine2000 in Optimem medium. For GFP-VEL1, 20 ng vector was topped up with 80 ng empty vector. After 5 h transfection, cells were changed into DMEM full medium and incubated a further 17 h for GFP-VEL1 and 24 h for GFP-VIN3 before FRAP analysis. Live cells were analyzed in a heated stage incubator at 37°C. Individual puncta or regions containing puncta were measured twice (5s interval) before bleaching, and the average of these values was set as 100%. These regions (ROI, region of interest) were subsequently bleached by a laser burst at 488 nm (Zeiss 710 confocal). Recovery of puncta was monitored by acquisition of images every 5 s for a total of three minutes. Only ROI where the puncta remained in the boxed area were considered for analysis. All error bars are represented as mean ± SEM for 6 independent experiments.

#### CoIP assays

HEK293T cells were seeded on poly-L-lysine coated plates at ∼70% confluency and transfected with a DNA:PEI (1:3.5) mixture after cells had attached. For GFP-VIN3, GFP-VEL1 and GFP-VRN5 coIPs, one 6-well plate per coIP was used. Cells were lysed ∼18 h post-transfection in 20 mM Tris pH 7.4, 200 mM NaCl, 10% glycerol, 5 mM NaF, 2 mM Na_3_VO_4_, 1 mM EDTA, 0.2% Triton X-100, EDTA-free protease inhibitor cocktail (Roche). Lysates were cleared by centrifugation (16,100 *g*, 10 min), and supernatants were incubated with GFP-trap (Chromotek) for 90 min at 4°C on an over-head tumbler. Immunoprecipitates were washed 4x in lysis buffer and eluted by boiling in lithium dodecyl sulfate (LDS) sample buffer for 10 min. Input and coIP fractions were separated by polyacrylamide gel electrophoresis (SDS-PAGE), blotted onto polyvinylidine difluoride (PVDF) membranes, checked for equal loading by Ponceau staining and processed for Western blotting with appropriate antibodies. Primary and secondary antibodies were diluted 1:5000 in phosphate-buffered saline (PBS), 0.05% Tween 20 and 5% milk powder. Blots were washed with PBS containing 0.05% Tween 20 and developed on film with ECL Western Blotting Detection Reagent.

#### Plant strains

The non-transgenic wt *Arabidopsis thaliana* strain Col-*FRI*^*SF2*^ and the *vin3-1 FRI* mutant have been described previously (Sung and Amasino, 2004).

#### Plant sampling

Samples were generated from leaves collected at three time points from wt and RI>DD VIN3-GFP transgenic lines: 32 days after sowing (NV); 6 weeks cold-treatment (6WT0); 10 days after the return to warm (6WT10). For VIN3-GFP RI>DD, four leaf pools were generated per time point from 36 independent first-generation transgenic plants (1 leaf per plant) after selection at NV. For homozygous control plants, three pools were generated with 1 leaf per plant from a total of 6 plants.

#### RNA extraction from plants and RT-qPCR

RNA was extracted as described (Questa et al., 2016), using acidic phenol followed by lithium chloride precipitation. RNA was DNase treated with Turbo DNA Free DNase, then transcribed into cDNA with SuperScript Reverse Transcriptase IV (both Life Technologies) with the gene-specific reverse primers detailed below. qPCR was performed using SYBRGreen Master Mix II on a LightCycler 480 II (both Roche) with primer pairs: VIN3 qPCR 1F 5′-TGCTTGTGGATCGTCTTGTCA-3′ and VIN3 qPCR 1R 5′-TTCTCCAGCATCCGAGCAAG-3′, FLC F 5′-AGCCAAGAAGACCGAACTCA-3′ and FLC R 5′-TTTGTCCAGCAGGTGACATC-3′, UBC qPCR F 5′-CTGCGACTCAGGGAATCTTCTAA-3′ and UBC qPCR R 5′-TTGTGCCATTGAATTGAACCC-3′, PP2A F2 5′-ACTGCATCTAAAGACAGAGTTCC-3′ and PP2A R2 5′-CCAAGCATGGCCGTATCATGT-3′. Results were normalized to the geometric mean of two standard genes, PP2A (At1g13320) and UBC (At5g25760).

#### VIN3 constructs for plant transformation

The genomic pENTR-VIN3:VIN3-GFP construct has been described (Questa et al., 2016). Site-directed mutagenesis was used to generate the R556D/I575D mutation using the primers R556D_F (5′-CAAGAGTTTTAGGGAAGACTTCTTGACATGGTATAG-3′), R556D_R (5′-CTATACCATGTCAAGAAGTCTTCCCTAAAACTCTTG-3′), I575D_F (5′-GAGAAGTAAGAGTTGTGAAGGACTTTGTTGAGACGTTTATGG-3′), and I575D_R (5′-CCATAAACGTCTCAACAAAGTCCTTCACAACTCTTACTTCTC-3′). L/R reaction (Invitrogen) was then used to transfer the pVIN3:VIN3-GFP constructs to the binary vector pSLJ-DEST (based on pSLJ755I6) (Jones et al., 1992), which were transformed into the vin3-1 *FRI* mutant mediated by Agrobacterium C58 using the floral dip method. Transgene copy number in the T1 transformants was assayed by IDna Genetics (Norwich Research Park).

#### Microscopy of plant specimens

Confocal imaging of soil-grown Arabidopsis lateral root tips was performed on a Zeiss LSM780 confocal microscope using a 40x/1.2 water objective. GFP was excited at 488 nm and detected at 491–535 nm.

#### Plasmids for N. benthamiana transfections

The coding sequence of *VIN3* was amplified from plasmid template (Greb et al., 2007) and cloned into the binary vector pCAMBIA1300 by in-fusion cloning to create *p35S: GFP-VIN3*. To increase translation efficiency, the Ω 5′-leader sequence was introduced upstream of the GFP ATG by restriction-free cloning to generate the expression vector *p35S: Ω-GFP-VIN3*. Site-directed mutagenesis was then used to create R554A R556D, and I575D.

#### Cell-based assays in N. benthamiana leaves

Plasmids containing GFP-VIN3 and related mutants were transformed into *Agrobacterium tumefaciens* C58 (pGV3101) using electroporation. Agrobacteria containing the desired construct were co-infiltrated with the silencing suppressor P19 at OD600 0.05 into three-week-old *Nicotiana benthamiana* leaves. Confocal imaging of *N. benthamiana* leaves was performed on a Zeiss LSM780 confocal microscope using a 40x/1.3 oil objective and 3× zoom, excitation at 488 nm, detection at 491–535 nm. Images were acquired 24 h after infiltration.

To determine protein levels, *N. benthamiana* leaves were harvested four days after co-infiltration with agrobacteria and the silencing suppressor P19 at OD600 0.6. Ten leaf disks were ground for each sample, and protein was extracted by incubation for 30 min in 50 mM Tris pH 7.5, 150 mM NaCl, 1 mM EDTA, 10% glycerol, 0.5% NP-40, 1 mM Na_2_MoO_4_, 1 mM NaF, 1.5 mM Na_3_VO_4_, 5 mM DTT, EDTA-free protease inhibitor cocktail (Roche) and cleared by centrifugation. Proteins were separated by SDS-PAGE after boiling in LDS sample buffer for 15 min, blotted onto PVDF membranes, checked for equal loading by Ponceau staining (visualizing the large subunit of Rubisco) and processed for Western blotting. Antibodies were diluted (primary 1:1000, secondary 1:15,000) in Tris-buffered saline (TBS), 0.05% Tween 20 and 5% milk powder. Blots were washed with TBS containing 0.05% Tween 20 and developed on film with ECL Western Blotting Detection Reagent.

### Quantification and statistical analysis

#### Analysis of EM data

180–300 measurements were taken from inverse FFTs of five separate VIN3_VEL_ lattices. The mean average ±standard deviation was calculated in Excel (Microsoft Office Professional Plus, 2019).

#### VEL floater assay

SEM values were calculated in Prism V8.0 (GraphPad) from measurements taken in four independent experiments.

#### Statistical analysis

Statistical details of individual experiments can be found in the figure legends. All error bars are represented as mean ± SEM for 3–6 independent experiments, except for the experiment shown in Figure 7 where the error bars denote standard deviations (calculated from 3–4 independent leaf pools). Statistical significance was calculated in Prism V8.0 (GraphPad) by ANOVA test and denoted as ^∗^ = p < 0.0001 between indicated data points.

## Data Availability

• Coordinates and crystal structure factors have been deposited at the Protein DataBank, and NMR data have been deposited at the BMRB (https://bmrb.io/) database; they are publicly available as of the date of publication. Accession numbers are listed in the key resources table. • This paper does not report original code. • Any additional information required to reanalyze the data reported in this paper is available from the lead contact upon request.

## Supplemental information

Document S1. Figures S1–S7 and Table S1

Document S2. Article plus supplemental information
