# Supplementary material for: Head-to-tail polymerization by VEL proteins underpins cold-induced Polycomb silencing in flowering control
Source: Cell Rep. 2022 Nov 8;41(6):111607. doi: 10.1016/j.celrep.2022.111607 (PMC7614096; doi:10.1016/j.celrep.2022.111607)
Supplement: Document S1. Figures S1–S7 and Table S1 [file mmc1.pdf]

Cell Reports, Volume *41*

Supplemental information

Head-to-tail polymerization by VEL proteins underpins cold-induced Polycomb silencing in flowering control

Marc Fiedler, Elsa Franco-Echevarría, Anna Schulten, Mathias Nielsen, Trevor J. Rutherford, Anna Yeates, Bilal Ahsan, Caroline Dean, and Mariann Bienz

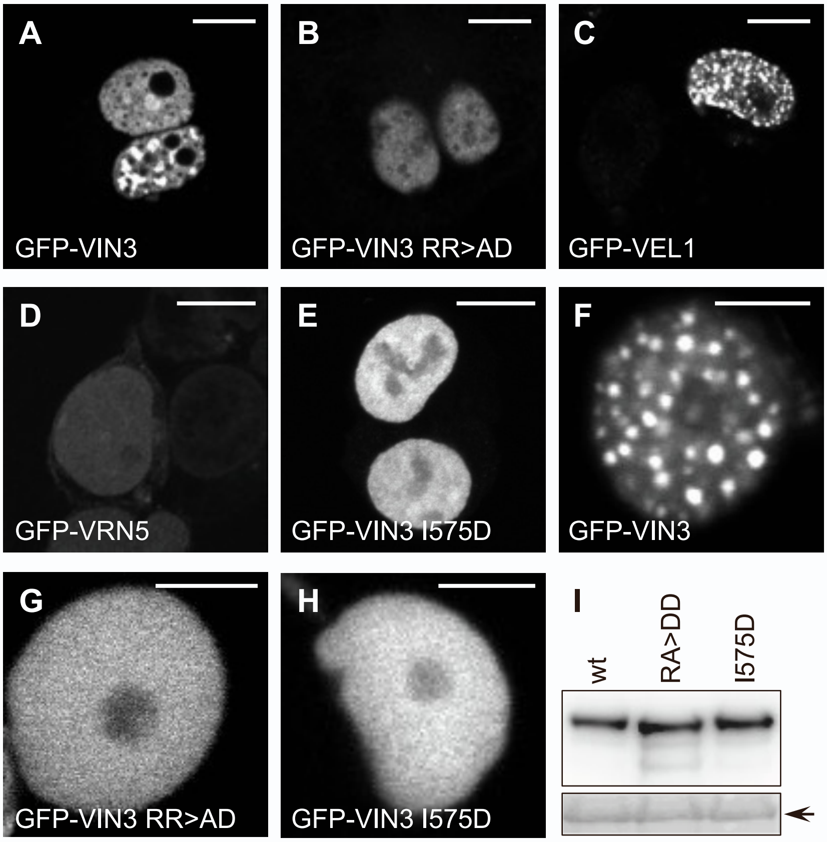

Supplementary Fig. 1 (related to Fig. 1)
VEL-dependent formation of nuclear condensates
(**A-E**) Representative confocal images of COS-7 (**A-C**) or HeLa (**D-E**) cells transfected with wt or mutant GFP-VIN3, GFP-VEL1 or GFP-VRN5, as indicated in panels; scale bars 10 µm. (**F-H**) Representative confocal images of *N. benthamiana* leaves infiltrated with wt or mutant GFP-VIN3, as indicated in panels; scale bars 5 µm. (**I**) Western blot showing comparable levels of wt and mutant GFP-VIN3 proteins; arrow, internal control (large subunit of Rubisco, visualized by Ponceau staining).

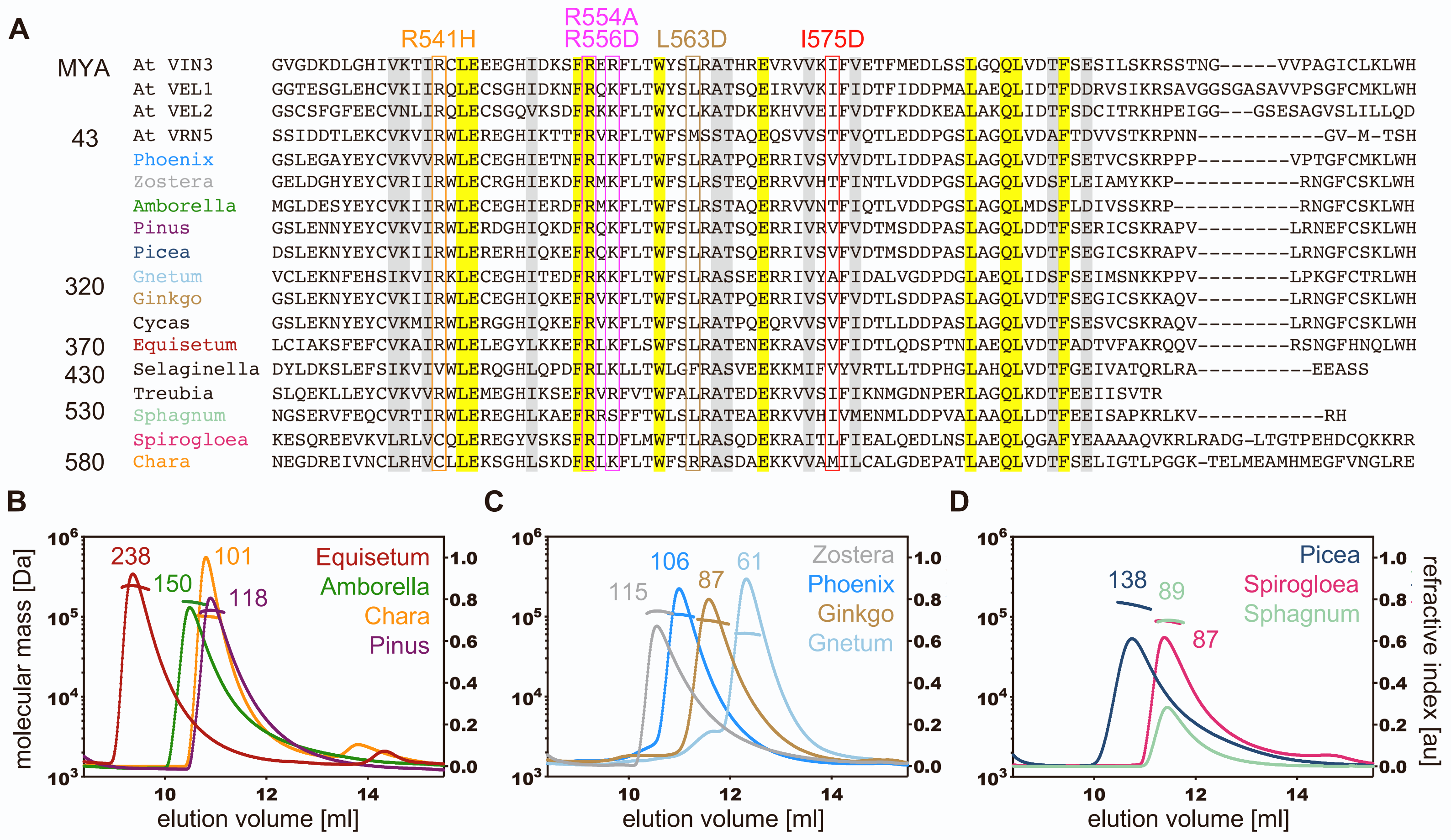

Supplementary Fig. 2 (related to Fig. 2)

Deep conservation of VEL domains in plants

(**A**) Sequence alignment of VEL domains from diverse plant species, as indicated (At, *Arabidopsis thaliana*; MYA, million years ago); point mutations disabling self-association of VIN3 are indicated above. (**B-D**) SEC-MALS of purified Lip-tagged VEL domains from various species as indicated in inset; *curves*, elution profiles; *line traces*, molar masses as derived from MALS.

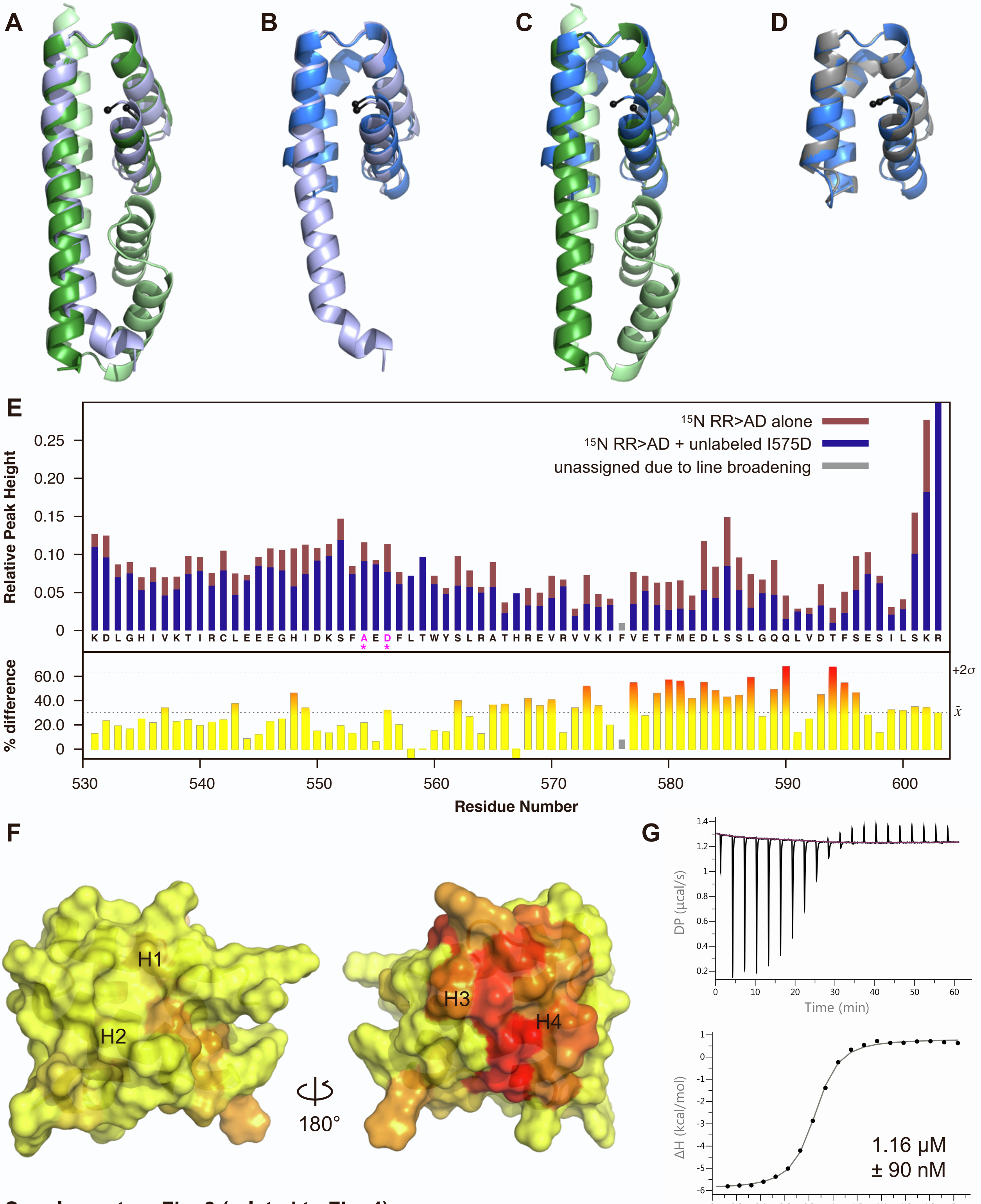

Supplementary Fig. 3 (related to Fig. 4)

Conserved VEL folds and confirmation of self-interacting surface by NMR

Overlays of ribbon diagrams of different VEL domains; *black balls*, N-termini; (**A**) VIN3VEL RR>AD (7O6U, *light blue*) and VIN3VEL R556D I575D (7O6T, *green*); RMSD 1.69 Å; (**B**) VIN3VEL RR>AD (7O6U, *light blue*) and VEL1VEL I664D (7O6W, *blue*), RMSD 1.63 Å; (**C**) VIN3VEL R556D I575D (7O6T, *green*) and VEL1VEL I664D (7O6W, *blue*), RMSD 1.77 Å; (**D**) VEL1VEL I664D (7O6W, *blue*) and VEL1VEL RK>AD I664D (7O6V, *gray*), RMSD 0.41 Å. (**E**) ^{15}N-labeled RR>AD probed with unlabeled I575D; top *panel*, relative peak heights from HSQC of unbound (*maroon*) versus HSQC of bound (*blue*); bottom panel, differences in percent. (**F**) Percent differences in (**E**) plotted onto the surface of the RR>AD NMR structural model, confirming that the tail surface as defined by crystallography also serves as the polymerisation interface in solution. (**G**) ITC profiles (top panel) of VIN3VEL RR>AD binding to VIN3VEL I575D. Fitted data (bottom panel) show a K*d* value of 1.16 μM for the dimeric interface.

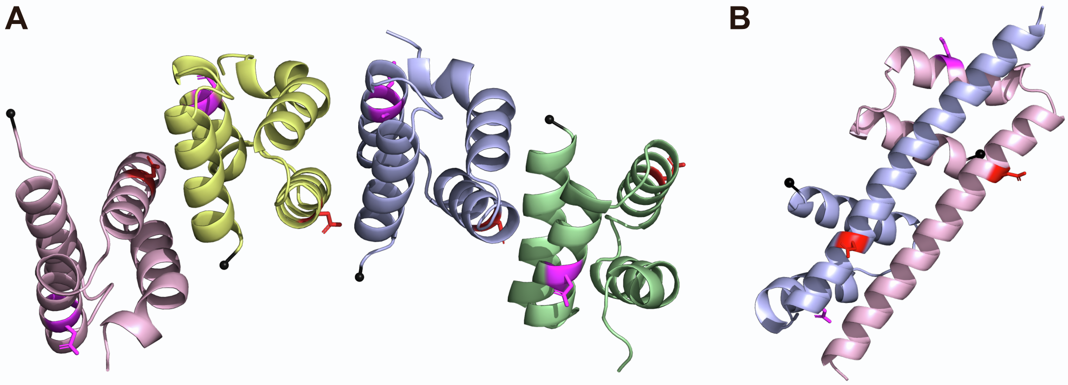

Supplementary Fig. 4 (related to Fig. 5)
Abnormal filaments of double-mutant VEL domains
Abnormal head-to-tail interactions in (**A**) VEL1$_{VEL}$ (R643A K645D I664D; 7O6V) or (**B**) VIN3$_{VEL}$ (R556D I575D; 7O6T) crystals bearing point mutations in both head and tail surfaces; *magenta*, R643A K645D (**A**), or R556D (**B**); *red*, I664D (**A**), or I575D (**B**).

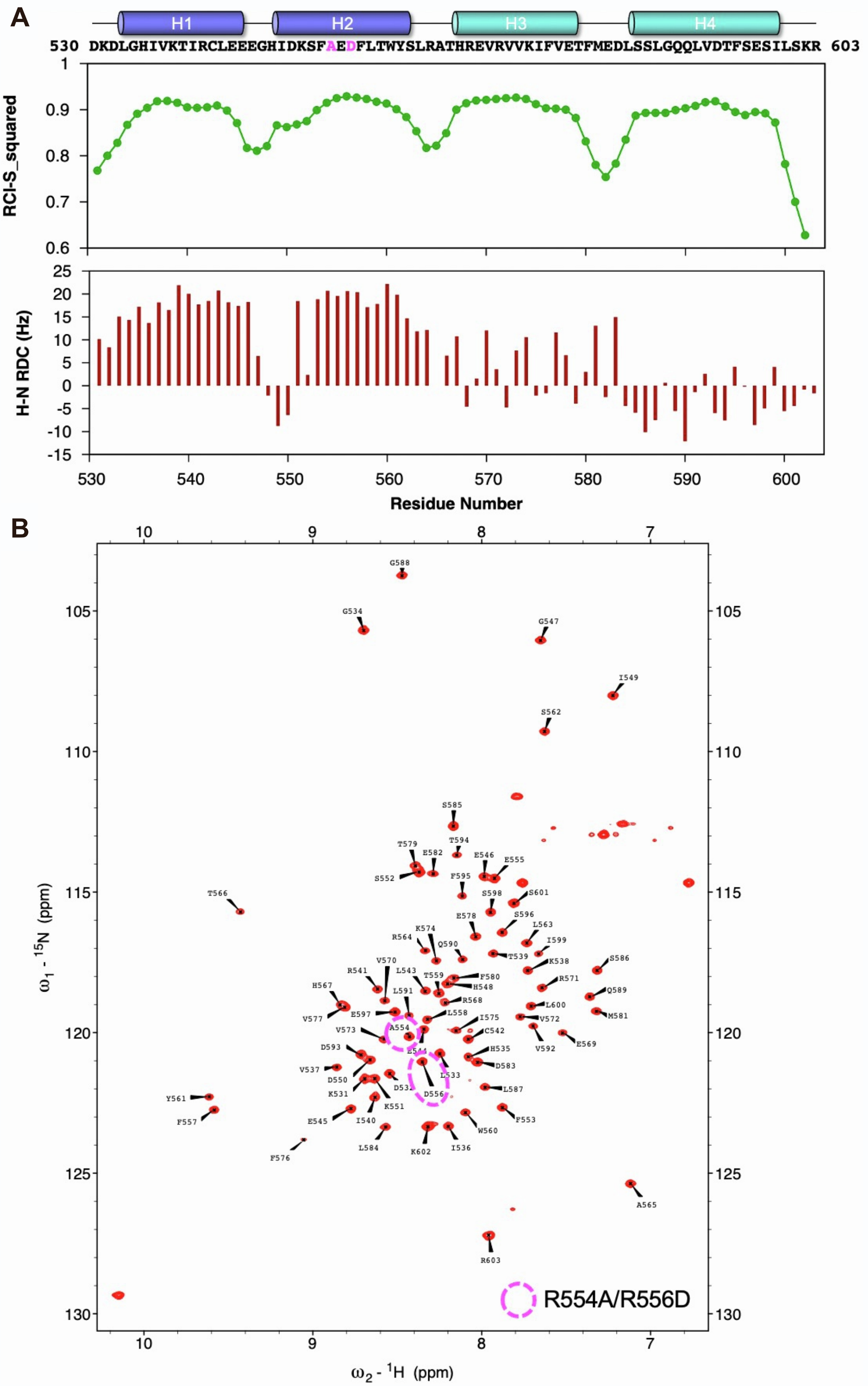

Supplementary Fig. 5 (related to Fig. 6)

NMR spectroscopy of VIN3$_{VEL}$ RR>AD

(**A**) VIN3$_{VEL}$ RR>AD sequence, with four α-helices above (*magenta*, R554A and R556D); RCI-S^2 showing the propensity of each residue (green dots) to be within a turn (<0.85) or an α-helix (>0.85); *bottom*, RDC plot further confirming 'H4 tucked under' conformation (see main text). (**B**) Full assignments of VIN3$_{VEL}$ 529-603, with RR>AD residues marked (*magenta*); spectrum acquired at 300 μM.

A

	VIN3_NMR	Z	VEL1_crystal	Z
1	**4GMQ** - RIBOSOME-BINDING DOMAIN OF ZUO1	6.1	6HPN - ANTIGEN, P35	6.4
2	6HPN - ANTIGEN, P35	6.0	**6CGH** - SOLUTION STRUCTURE OF THE FOUR-HELIX BUNDLE REGION OF HUMAN J-PROTEIN ZUOTIN	6.3
3	**5Y6O** - CRYSTAL STRUCTURE OF DAXX N-TERMINAL FOUR-HELIX BUNDLE DOMAIN (4HB) IN COMPLEX WITH ATRX	5.9	**4GMQ** - RIBOSOME-BINDING DOMAIN OF ZUO1	5.0
4	3TDW - GENTAMICIN RESISTANCE PROTEIN	5.2	**5Y6O** - CRYSTAL STRUCTURE OF DAXX N-TERMINAL FOUR-HELIX BUNDLE DOMAIN (4HB) IN COMPLEX WITH ATRX	5.0
5	**6CGH** - SOLUTION STRUCTURE OF THE FOUR-HELIX BUNDLE REGION OF HUMAN J-PROTEIN ZUOTIN	5.0	1OKS - RNA POLYMERASE ALPHA SUBUNIT	4.8
6	2IPC - PREPROTEIN TRANSLOCASE SECA SUBUNIT	4.9	3F7W - PUTATIVE FRUCTOSAMINE-3-KINASE	4.7
7	1OKS - RNA POLYMERASE ALPHA SUBUNIT	4.7	1K30 - GLYCEROL-3-PHOSPHATE ACYLTRANSFERASE	4.7
8	3F7W - PUTATIVE FRUCTOSAMINE-3-KINASE	4.7	6N9Y - NON-STRUCTURAL PROTEIN 1 OF BLUETONGUE VIRUS	4.6
9	**2LWX** - SOLUTION STRUCTURE OF THE C-TERMINAL PDR1-ACTIVATING DOMAIN OF THE J-PROTEIN ZUO1	4.6	3TDW - GENTAMICIN RESISTANCE PROTEIN	4.6
10	5LXJ - PHOSPHOPROTEIN	4.5	2IPC - PREPROTEIN TRANSLOCASE SECA SUBUNIT	4.6

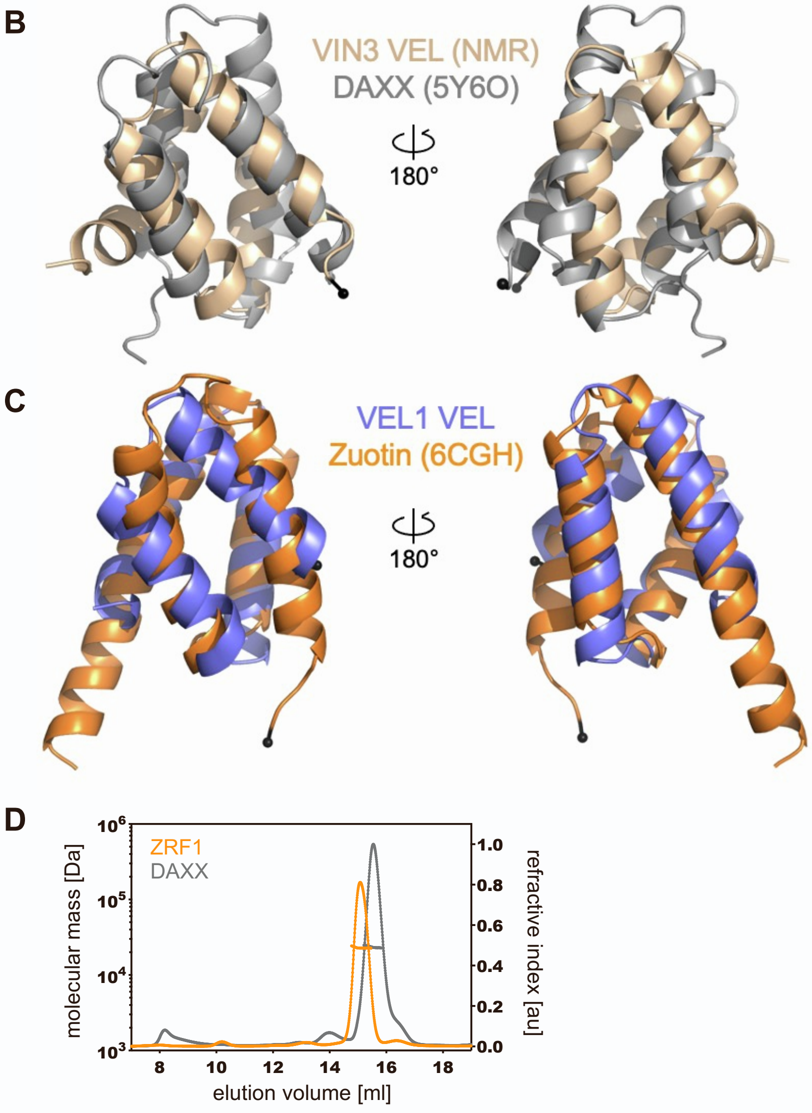

Supplementary Fig. 6 (related to Fig. 6)

VEL-related 4HB domains in DNAJ co-chaperones

(**A**) Table of top hits from DALI searches for VIN3$_{VEL}$ (*left*) and VEL1$_{VEL}$ (*right*), with 4HB from Zuotin (*orange*) and DAXX (*gray*) highlighted. (**B, C**) Overlays of (**B**) VIN3$_{VEL}$ RR>AD (*wheat*) with 4HB domain of DAXX (5Y6O; *gray*), RMSD 3.24 Å, (**C**) VEL1$_{VEL}$ (*blue*) with 4HB domain of Zuotin (6CGH; *orange*), RMSD 2.69 Å; *black balls*, N-termini. (**D**) SEC-MALS of human DAXX (*gray trace*) and human ZRF1 (*orange trace*), both corresponding to monomers; void volume of column at 8 ml.

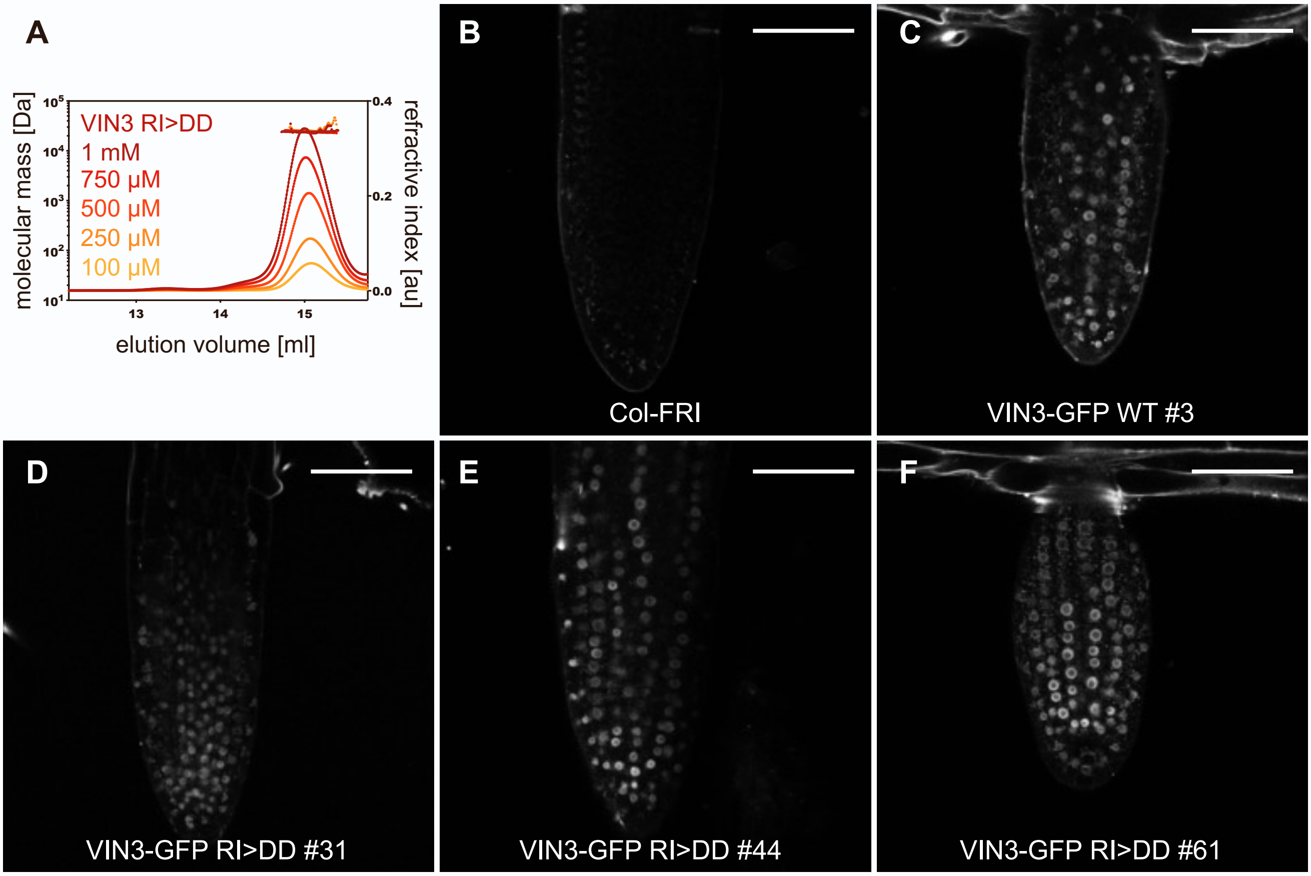

Supplementary Fig. 7 (related to Fig. 7)

Nuclear localization of wt and RI>DD mutant VIN3-GFP

(**A**) SEC-MALS of purified Lip-VIN3$_{VEL}$ RI>DD, at increasing concentrations as indicated in panels; *line traces*, molar masses as derived from MALS. Highest concentration is still monomeric (calculated 23.4 kDa, observed 24 kDa); void volume of column at 8 ml. (**B-F**) Confocal images of Arabidopsis root tips expressing wt VIN3-GFP (homozygous) or polymerization-deficient RI>DD mutant (independent first generation transgenic lines) at 6WT0. Scale bar 50 µm. Transgene copy numbers in individual RI>DD lines are 3 (#31), 11 (#44) and 3 (#61). Note that all images were taken with the same microscopy settings and on the same day from the same individual plants as those shown in main Fig. 7 whose flowering is delayed compared to wt VIN3-GFP, revealing comparable levels of fluorescence in the nuclei of these plants.

	**VEL1_VEL**		**VIN3_VEL**		
**PDB accession codes**	**I664D**	**R643A K645D I664D**	**I575D**	**R554A R556D**	**R556D I575D**
	**7O6W**	**7O6V**	**7OQV**	**7O6U**	**7O6T**
**Crystal data**					
Wavelength (Å)	0.97950	0.97950	0.97950	0.97950	0.97950
Resolution (Å)	27.47 – 2.64 (2.77 – 2.64)	29.03 – 2.5 (2.6 – 2.5)	29.07 – 2.4 (2.49 – 2.4)	28.82 – 1.84 (1.88 – 1.84)	29.47 – 2.02 (2.07 – 2.02)
Space group	$P\ 6_3$	$P\ 2_1\ 2_1\ 2_1$	$P\ 2_1\ 2_1\ 2_1$	$I\ 4_1$	$P\ 2_1\ 2_1\ 2_1$
Unit cell dimensions					
a, b, c (Å)	79.26, 79.26, 59.96	52.07, 58.05, 119.99	31.5, 91.27, 98.41	40.75, 40.75, 83.96	31.42, 51.81, 85.01
α, β, γ (°)	90, 90, 120	90, 90, 90	90, 90, 90	90, 90, 90	90, 90, 90
Total reflections	239198 (22200)	338001 (38598)	101791 (10799)	82212 (4592)	349471 (22221)
Unique reflections	6335 (769)	13183 (1481)	11740 (1191)	5941 (354)	9677 (677)
Multiplicity	37.8 (29.2)	25.6 (26.1)	8.7 (9.1)	13.8 (13)	36.1 (32.8)
Mean I/σ (I)	37.0 (3.9)	9.6 (2.7)	13.3 (2.8)	39.2 (3.9)	36.2 (4.6)
R merge (%)	6.8 (104.5)	24.4 (141.3)	9.8 (76.2)	4.5 (66.5)	7.0 (87.7)
$CC_{1/2}$	1.00 (0.84)	0.99 (0.84)	1.00 (0.91)	1.00 (0.91)	1.00 (0.93)
Completeness (%)	98.8 (92)	100 (100)	100 (100)	99.7 (95.1)	99.7 (96.5)
Complexes in A.U.	2	4	4	1	2
**Refinement**					
Resolution	27.47 – 2.64	29.03 – 2.5	29.07 – 2.4	28.82 – 1.84	29.47 – 2.02
Number of reflections	6281	13134	11698	5913	9609
R_{work} / R_{free} (%)	21.16 – 24.92	21.36 – 24.07	22.34 – 26.95	22.13 – 24.44	20.24 – 23.66
**Nº of atoms**	1151	2308	2357	604	1223
Protein	1140	2299	2319	588	1173
Ligand	5	-	-	-	1
Water	6	9	38	16	49
**Average B Factors** (Å^2)					
Wilson/overall	80.42	53.72	45.12	38.71	41.25
Protein	66.86	45.62	60.33	33.78	34.11
Ligand	161.50	-	-	-	52.82
Water molecules	79.40	53.22	52.33	48.46	50.39
All atoms	67.33	45.65	60.20	34.17	34.78
**RMSDs deviations**					
Bonds lengths (Å)	0.006	0.008	0.007	0.010	0.009
Bond angles (°)	1.01	1.16	1.53	1.50	1.22
**Ramachandran plot Statistics** (%)					
Favored regions	97.76	100	98.89	100	100
Allowed regions	2.24	0	1.11	0	0
Disallowed regions	0	0	0	0	0

Table S1

Crystal data collection and refinement statistics
